# Ultra-low volume intradermal administration of radiation-attenuated sporozoites with the glycolipid adjuvant 7DW8-5 completely protects mice against malaria

**DOI:** 10.1038/s41598-024-53118-9

**Published:** 2024-02-04

**Authors:** Felicia N. Watson, Melanie J. Shears, Anya C. Kalata, Caroline J. Duncombe, A. Mariko Seilie, Chris Chavtur, Ethan Conrad, Irene Cruz Talavera, Andrew Raappana, D. Noah Sather, Sumana Chakravarty, B. Kim Lee Sim, Stephen L. Hoffman, Moriya Tsuji, Sean C. Murphy

**Affiliations:** 1 Graduate Program in Pathobiology, Department of Global Health, University of Washington, Seattle, WA 98109 USA; 2 Department of Laboratory Medicine and Pathology, University of Washington, Seattle, WA 98195 USA; 3 Center for Emerging and Re-emerging Infectious Diseases (CERID), University of Washington, Seattle, WA 98109 USA; 4 Center for Global Infectious Disease Research, Seattle Children’s Research Institute, Seattle, WA 98109 USA; 5 Sanaria Inc., 9800 Medical Center Drive, Suite A209, Rockville, MD 20850 USA; 6 Aaron Diamond AIDS Research Center, Division of Infectious Diseases, Department of Medicine, Columbia University Irving Medical Center, New York, NY 10032 USA; 7 Department of Microbiology, University of Washington, Seattle, WA 98109 USA; 8 Washington National Primate Research Center, University of Washington, Seattle, WA 98109 USA; 9 Department of Laboratories, Seattle Children’s Hospital, Seattle, WA 98105 USA

**Keywords:** Translational research, Malaria, Adjuvants, DNA vaccines, Live attenuated vaccines

## Abstract

Radiation-attenuated sporozoite (RAS) vaccines can completely prevent blood stage *Plasmodium* infection by inducing liver-resident memory CD8^+^ T cells to target parasites in the liver. Such T cells can be induced by ‘Prime-and-trap’ vaccination, which here combines DNA priming against the *P. yoelii* circumsporozoite protein (CSP) with a subsequent intravenous (IV) dose of liver-homing RAS to “trap” the activated and expanding T cells in the liver. Prime-and-trap confers durable protection in mice, and efforts are underway to translate this vaccine strategy to the clinic. However, it is unclear whether the RAS trapping dose must be strictly administered by the IV route. Here we show that intradermal (ID) RAS administration can be as effective as IV administration if RAS are co-administrated with the glycolipid adjuvant 7DW8-5 in an ultra-low inoculation volume. In mice, the co-administration of RAS and 7DW8-5 in ultra-low ID volumes (2.5 µL) was completely protective and dose sparing compared to standard volumes (10–50 µL) and induced protective levels of CSP-specific CD8^+^ T cells in the liver. Our finding that adjuvants and ultra-low volumes are required for ID RAS efficacy may explain why prior reports about higher volumes of unadjuvanted ID RAS proved less effective than IV RAS. The ID route may offer significant translational advantages over the IV route and could improve sporozoite vaccine development.

## Introduction

The global burden of malaria remains unacceptably high with an estimated 247 million infections and 619,000 deaths in 2021^1^. Many clinical malaria cases are concentrated in sub-Saharan Africa and are caused by *P. falciparum* (Pf), which is transmitted through the bites of infectious female *Anopheles* mosquitoes. Several pre-erythrocytic and erythrocytic vaccines target Pf and can provide varying degrees of protection against infection, clinical disease, and death (reviewed in^2,3^). However, the only vialed vaccines to routinely induce sterile protection against Pf challenge in humans are live-attenuated whole sporozoite (spz) vaccines (*i.e.*, Sanaria PfSPZ Vaccine and PfSPZ-CVac^4–12^). These are aseptic, purified, cryopreserved spz vaccines that induce both humoral and cellular immune responses^11^. Antibodies are mainly induced by the immunodominant circumsporozoite protein (CSP) antigen, and these antibodies can bind to spz to block hepatocyte invasion^4,13,14^. Although high titers of CSP-binding antibodies alone can confer high levels of protection^15,16^, induction of CD8^+^ T cells, specifically liver-resident memory CD8^+^ T (Trm) cells, appears to be critical for reliable and durable sterile protection^17,18^.

To simplify and improve whole spz vaccination, we developed a two-step heterologous vaccine strategy called prime-and-trap^19,20^. This vaccine combines priming with a nucleic acid-based vaccine in the periphery (*e.g.*, skin) followed by expression of the cognate antigen in the liver through spz- or other vehicle-mediated delivery. In its first generation, prime-and-trap was based on skin priming using plasmid DNA encoding the *P. yoelii* (Py) rodent malaria CSP antigen followed by a single intravenous (IV) dose of cryopreserved radiation attenuated spz (cryo-RAS) to direct and “trap” the activated and expanded CD8^+^ T cells in the liver. This strategy induced robust CSP-specific CD8^+^ Trm responses in the liver and conferred durable sterile protection in this rodent malaria model for at least four months^20^. However, it was unclear whether the RAS dose must be strictly administered IV. This question is of substantial interest since success with non-IV administration routes could simplify the translational feasibility of spz vaccines.

Intradermal (ID) administration of RAS is an attractive alternative to IV administration since it attempts to mimic the natural route of exposure via mosquito bite. Moreover, the skin is accessible, patrolled by antigen presenting cells (APCs), and compared to other routes, can be dose-sparing^21–23^. Unfortunately, previous attempts at ID RAS administration (ID-RAS) in mice or humans were ineffective—there was substantially higher vaccine efficacy following IV RAS administration (IV-RAS) than after ID^11,24–27^. In prior studies, the amount of vaccine spz delivered to the liver as measured by total liver parasite burden was reduced after ID as compared to IV administration, and this difference was implicated as a primary reason for the failure of the ID route^28,29^. Other studies have also suggested that the reason for ID spz administration failure may be due to the more tolerogenic environment of the skin, which could ultimately lead to more regulatory immune responses in the liver^25,30^. However, most of these studies used standard ID injection volumes (10–50 µL), which do not mimic the ultra-low volumes delivered by probing mosquitoes^31,32^, nor facilitate efficient exit of spz from the skin, since spz must move by contact-dependent motility^33,34^. Based on the available data, and recognition of this unique biology and motility requirements of spz, we hypothesized that two key aspects of RAS administration are critical for effective ID vaccination: 1) the injection volume must be compatible with the contact-dependent motility of the spz, and 2) the tolerogenic skin immune environment must be overcome.

In this study, we used the Py rodent malaria model to determine if ID-RAS can replace IV-RAS as the trapping component of the prime-and-trap vaccine. As ID-RAS are known to be less immunogenic than IV-RAS, we investigated if we could improve the efficacy of ID-RAS trapping by reducing the volume and/or co-administering RAS with the glycolipid adjuvant, 7DW8-5. 7DW8-5 is a synthetic glycolipid adjuvant that was selected for this vaccine approach because it potently activates invariant natural killer T (*i*NKT) cells to preferentially induce Th1 cytokines (*e.g.*, IFN-γ), inducing a cascade of immune cell activation including CD8^+^ T cells (reviewed in^35^). We showed that mice primed with DNA encoding the PyCSP antigen administered via gene gun followed by trapping with 7DW8-5-adjuvanted ID-RAS (ID-RAS + 7DW8-5) are highly protected against Py spz challenge. We also showed that reducing the volume used for ID-RAS to an ultra-low volume of 2.5 µL is dose-sparing and provides sterile protection for at least four months. Furthermore, these modifications have the potential to improve RAS-only vaccination in addition to prime-and-trap. Overall, we demonstrate that ID-RAS is as protective as IV-RAS when co-administered with a potent adjuvant in an ultra-low volume and may provide an alternative non-IV route for spz vaccination.

## Materials and methods

### Mice

Female 4–6 week-old BALB/cJ mice were purchased from Jackson Laboratories (Bar Harbor, ME) and housed at the University of Washington in an Institutional Animal Care and Use Committee (IACUC)-approved animal facility. All mice were used under an approved IACUC protocol (4317–01 to SCM) and in accordance with relevant guidelines and regulations. All methods are reported in accordance with ARRIVE guidelines.

### DNA vaccination by gene gun

The Py circumsporozoite protein (CSP) DNA vaccine plasmids were constructed in the pUb.3 vector and co-administered with *Escherichia coli* heat-labile toxin (LT)-encoding plasmid adjuvant as described^19,36–38^. The PyCSP-minigene encodes the SYVPSAEQI epitope and the PyCSP plasmid encodes the full-length CSP protein without the major repeat region. Supplementary Fig. 1 details amino acid sequences and agarose gel restriction digest plasmid validation for all PyCSP vaccines. All plasmid stocks were Sanger sequenced (GeneWiz Inc.) before use. Gene gun DNA vaccine cartridges were constructed as previously described^20,37^. Mice were vaccinated on a shaved abdomen using a PowderJect-style gene gun by priming using two cartridges per day on Days 0 and 2 (0.5 µg DNA per cartridge). This method of priming with PyCSP/LT-encoding plasmids via gene gun is referred to as ggCSP.

### Cryopreserved irradiated spz vaccination

Cryopreserved Py wild-type (WT) 17XNL (cryo-RAS) were radiation-attenuated (100 Gy by C0-60), purified, vialed, and produced by Sanaria Inc. (Rockville, MD)^11,39^. The vials were shipped to Seattle and stored in vapor phase liquid nitrogen per manufacturer recommendations. Cryo-RAS were thawed in a 37°C water bath for 30 s, diluted in Schneider’s insect media (Gibco, Thermo Fisher Scientific), and administered within 30 min of thawing. Spz counts were confirmed on a hemocytometer within one hour of injection. Figure legends specify the dose, volume, route, and number of injections for each experiment.

### Freshly-dissected spz production and challenge

Female *Anopheles stephensi* mosquitoes infected with wild-type *P. yoelii* 17XNL (Py WT) were reared at Seattle Children’s Research Institute (Seattle, WA). Fresh spz were obtained by salivary gland dissection 14–18 days post-infection followed by Accudenz gradient purification as described^40^. Heat-killed spz (HK-spz) were generated by incubating Py WT spz in a 55°C water bath for 30 min. All spz were diluted in Schneider’s insect media for administration. Figure legends specify the dose, volume, route, and number of injections for each experiment. For all spz challenge administrations, 1 × 10^3^ freshly-dissected Py WT spz in 100 µL were injected retro-orbitally (RO) IV. Blood stage protection after spz challenge was assessed by Giemsa (Sigma-Aldrich) stained thin blood smear microcopy on Days 3–14 post-challenge. Mice were deemed protected if blood smears remained negative for parasites up to Day 14.

### Intradermal and intravenous spz injections

ID injections in standard volumes (STV) of 10–50 µL were administered with a BD Veo Insulin Syringe with Ultra-Fine needle 6mm x 31G 3/10 mL/cc (#324909). STV injections were administered in two ID injections per dose on the lower back near the base of the tail. Ultra-low volume (ULV) ID injections of 2.5 µL were administered with a 10 µl Sub-microliter injection syringe (World Precision Instruments, Inc #NANOFIL) and a 36G Beveled needle (World Precision Instruments, Inc #NF36BV). ULV injections were administered in two ID injections per dose on the left rear footpad. IV injections were all administered RO in 100 µL with an Exel International Insulin Syringes with a 29G permanently attached needle. Supplementary Fig. 2 diagrams the locations of all ID and IV injections.

### Glycolipid adjuvant preparation

7DW8-5 powder previously made under Good Manufacturing Practice (GMP) conditions was reconstituted in DMSO and prepared for injection as described^20^. 7DW8-5 or DMSO vehicle control was mixed with the cryo-RAS vaccines immediately before administration. All mice received 2 µg of 7DW8-5 adjuvant per immunization.

#### ELISA

Interferon-γ (IFN-γ) or IL-4 cytokine levels were determined by commercial ELISA kit according to manufacturer’s instructions (BioLegend, San Diego, CA; #430801 and #431104). Blood was collected into tubes containing EDTA and then plasma was isolated and frozen. For liver tissue, half of the liver was excised, weighed, and pulverized by bead beating in 3 mL lysis buffer (phosphate-buffered saline (PBS), 1:100 Pierce protease inhibitor (Thermo Fisher Scientific, # A32953), 0.05% Triton X-100). Homogenized samples were centrifuged at 16,000 × g for 10 min at 4°C. Supernatant was collected and frozen. All samples were diluted in the kit assay diluent, and absorbance was read on the CLARIOstar Plus plate reader (BMG Labtech, Germany) according to kit instructions. Standard curves and cytokine concentrations were calculated in Microsoft Excel.

PyCSP binding antibodies in mouse serum were determined by direct ELISA as previously described^41^. Blood was collected via submental bleed; serum was isolated and frozen. All serum samples were heat inactivated for 30 min at 56°C and centrifuged at 17,000 × g for 10 min prior to ELISA analysis. 50 ng per well recombinant PyCSP was plated in in 0.1M NaHCO_3_, pH 9.5, and incubated overnight at room temperature. Serum was diluted over a range of 1:50 to 1:109,350, and binding was detected with goat anti-mouse IgG Fc-HRP (Southern Biotech, #1013-05). Absorbance at 450 nm was determined with the BioTek ELx800 reader.

### Depletion/blocking antibodies

For CD1d and CD8 depletion/blocking studies, mice were injected intraperitoneally (IP) with 100 μg of anti-mouse CD1d (BioXcell, Lebanon, NH; #BE0000; Clone: 19G11) or 500 μg of anti-mouse CD8 (BioXcell, #BE0061; Clone: 2.43) 24 h before challenge. Matched isotype controls were used at the same concentration respectively (BioXcell, #BE0088; Clone:HRPN (CD1d) or #BE0090; Clone: LTF-2 (CD8)). Additional animals were used to validate the depletion doses and schedule used for these studies (Supplementary Fig. 7). The depletion dose for CD8 was validated by whole blood leukocyte flow cytometry and CD1d dose was validated by plasma IFN-γ ELISA, as described below.

For CD8 depletion confirmation by flow cytometry, blood was collected via submental bleed into tubes containing EDTA 24 h post CD8 depletion antibody or isotype injection. Whole blood was then resuspended in ammonium-chloride-potassium lysis buffer for 2–3 min to lyse red cells. The reaction was quenched with MACS buffer (PBS, 1 mM EDTA, 0.5% fetal bovine serum (FBS)). The final cell pellet containing whole blood leukocytes was resuspended in MACS buffer, blocked, stained, and fixed for flow cytometry as described below. The following Abs were used to assess CD8 cell depletion validation: live/dead dye-NIR, CD3e-BUV395, B220-BV711, CD4–Alexa Fluor, CD8a-BV421. Detailed information on flow reagents in Supplementary Table 1. Cell count per 100 μL blood was calculated based on known starting volume of mouse blood to normalize data. Flow cytometry was conducted on the LSRII instrument (BD Biosciences), and data were analyzed with FlowJo version 10.7.1 (BD Biosciences). For CD1d blocking confirmation, IFN-γ induced by 7DW8-5 was measured by ELISA. At 24 h post CD1d or isotype depletion, 7DW8-5 was injected by the IV route. Six hours later, blood was collected (as described above), plasma was isolated, and IFN-γ cytokine levels were analyzed by ELISA was described above.

### RAM2 spz-invasion blocking antibodies

RAM2 monoclonal antibodies were kindly provided by Noah Sather at Seattle Children’s Research Institute. RAM2 antibodies were produced and purified as described^41^. For spz-invasion studies, mice were injected IP with 150 μg of RAM2 or matched isotype control 24 h before RAS immunization. Two hours post immunization, blood was collected via submental bleed and serum was isolated to quantify the amount of antibody circulating via ELISA, using RAM2 as a standard curve as previously described^42^. Serum was serially diluted over a range of 1:25 to 1:1,476,225 and binding was determined as described above with goat anti-mouse IgG-HRP (Southern Biotech, #1015–05). Standard curves for RAM2 were generated by nonlinear regression (log[agonist] vs response[three parameters]) in GraphPad Prism (San Diego, CA). Serum antibody concentrations were quantified by interpolating the average values from three different dilutions along the sample binding curve to the corresponding standard curves and multiplying by the dilution factor to determine the final concentration.

### Parasite burden reverse transcription polymerase chain reaction (RT-PCR)

To quantify liver burden, half of the liver was excised, pulverized by bead beating into NucliSENS lysis buffer (bioMérieux), and nucleic acid was extracted as previously described^20,43^. RNA was subjected to RT-PCR with the SensiFAST™ Probe Lo-ROX Kit (Bioline, London, UK) using a mouse GAPDH RT-PCR assay (IDT Inc, Coralville, IA) multiplexed with a Pan-*Plasmodium* 18S rRNA assay on a QuantStudio 5 real-time PCR machine (Thermo Fisher Scientific) as described^44^. *Plasmodium* 18S rRNA copy numbers per reaction were determined using a custom lot of quantified Armored RNA encoding full-length *Plasmodium* 18S rRNA (Asuragen, Austin, TX). To quantify popliteal draining lymph node (PO dLN) burden, the left PO dLNs were excised and pooled with alike PO dLN from the same group. Pooled PO dLNs were pulverized by bead beating in NucliSENS lysis buffer and processed for RT-PCR as described above.

### Liver hepatic mononuclear cells (HMNCs) isolation and flow cytometry

Liver Hepatic mononuclear cells (HMNCs) were isolated by mechanical dissociation and Percoll density gradient as previously described^19,45^. Briefly, livers were excised, mashed into a single cell suspension, and intrahepatic lymphocytes were isolated. Final liver lymphocyte pellets were transferred to a V-bottom 96-well plate for blocking, staining, and fixing for flow cytometry. All antibodies and staining conditions were as previously described^19,20^ and reagents are listed in Supplementary Table 1. Representative gating strategy is shown in Supplementary Fig. 6. Flow cytometry was conducted on the LSRII instrument (BD Biosciences), and data were analyzed with FlowJO version 10.10.0 (BD Biosciences).

### Ex vivo* IFN-γ ELISPOT*

PyCSP peptide (SYVPSAEQI) was synthesized by Genemed Synthesis and reconstituted in DMSO. Mouse IFN-γ ELISPOT (eBioscience) was conducted by stimulating cells with CSP peptide (or DMSO vehicle control) at 1 µg/ml for 18 h at 37°C and developed following manufacturer guidelines as reported previously^19,46^. The number of spot-forming units (SFU) in each well was calculated using an ImmunoSpot 5.1 Analyzer (Cellular Technology Limited, OH). SFU were normalized to DMSO control wells and SFU per million cells were reported.

### nCounter® gene expression

Gene expression analysis was performed using the NanoString nCounter® Mouse Host Response Panel. Liver samples were prepared as described above for RT-PCR with n = 3 mice per group. Total RNA was extracted on the EasyMag system (bioMérieux) and the concentration was estimated with Nanodrop (Thermo Fisher Scientific). RNA (100 ng) was prepared for gene expression analysis at the Fred Hutchinson Cancer Research Center Genomics & Bioinformatics Core (Seattle, WA). Briefly, RNA samples were mixed with biotinylated capture and florescent reporter probes that were hybridized at 65°C for 12–16 h. Hybridized samples were run on the NanoString nCounter® Mouse Host Response Panel using the recommended manufacturer protocol. After data collection, the nCounter®. RCC files were imported into nSolver Analysis Software 4.0 for review of quality control metrics, and the panel of housekeeping genes and positive controls was used to compute the normalization factor. Further data analysis was performed in RStudio version 2022.02.01 + 461 with R version 4.1.3. The normalized count matrix was evaluated for outliers using principal component analysis and no outliers were identified. Log_2_ transformed normalized counts per million were assessed for differential expression for ~ 0 + vaccine using limma version 3.50.3^47^. Pairwise contrasts were performed for each vaccine group (IV-RAS, ID-RAS, ID-RAS + 7DW8-5) and control (ggCSP only). Significant genes were defined at FDR < 0.05 with BH correction and an absolute log_2_ fold change > 1 (Supplementary Files 1, 2). Selected pathways from MSigDB hallmark and KEGG collections^48,49^ were utilized to visualize differentially expressed genes.

### Statistics

Comparisons of parasite burden RT-PCR, flow cytometry, and ELISPOT groups were done using non-parametric Kruskal–Wallis one-way analysis of variance with Dunn’s multiple comparisons test. ELISA data was analyzed with non-parametric Mann–Whitney test unless otherwise specified in the figure legend. Protection data was evaluated using Fisher’s exact test. All groups were compared against the ggCSP prime and 2 × 10^4^ IV RAS trap positive control as a benchmark. Error bars in figures are reported as standard deviation (SD) of the mean with individual mouse samples shown if applicable. All *p*-values and individual experiment statistics are listed in corresponding figure legends. Statistical significance was defined as *p* < 0.05. Prism GraphPad Prism 9.1.2 Software (San Diego, CA) was used for all calculations, unless noted otherwise.

## Results

### Glycolipid adjuvant 7DW8-5 potentiates prime-and-ID RAS trap vaccination

**Figure 1:**
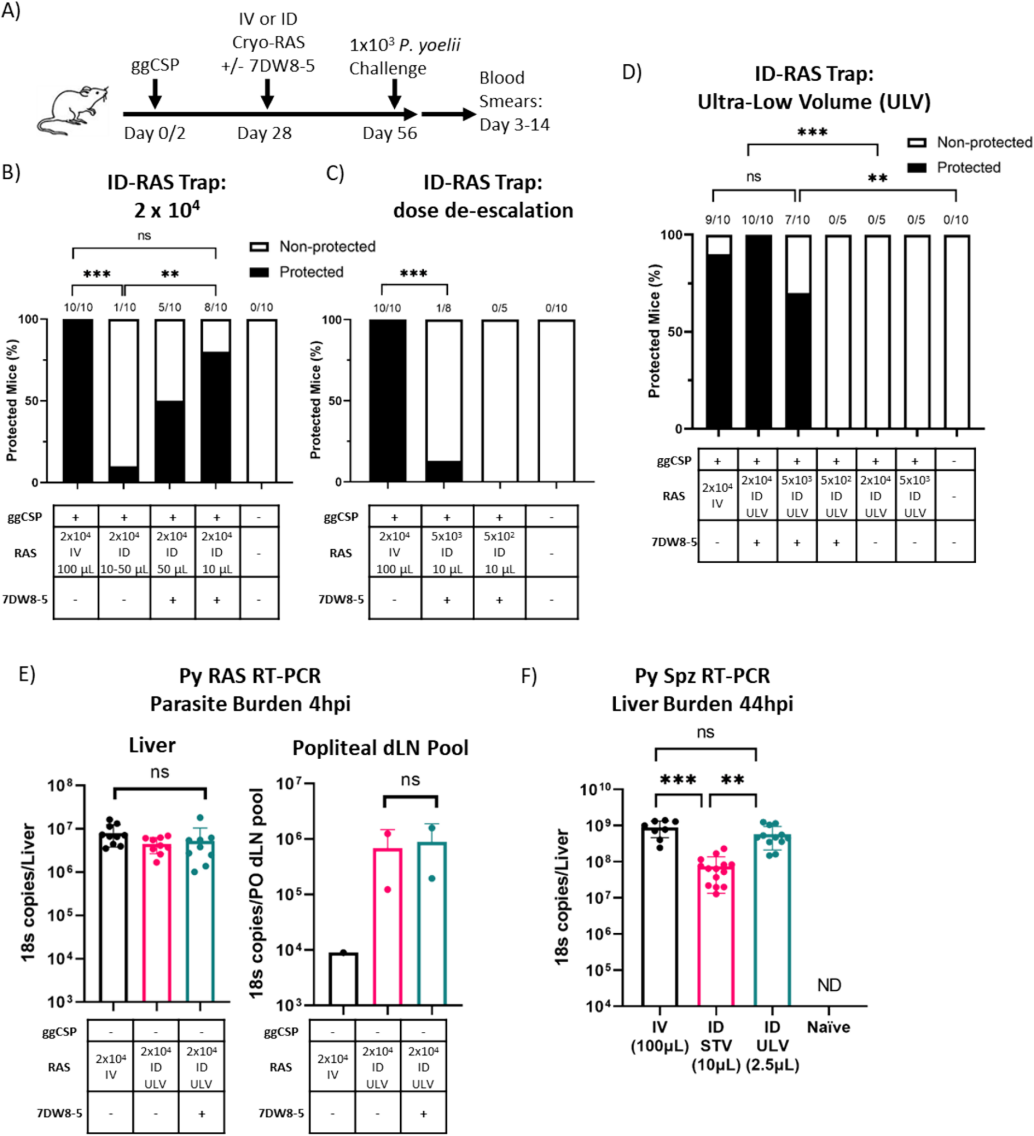
Prime-and-ultra-low volume 7DW8-5-adjuvanted ID-RAS trap completely protects mice against Py spz challenge. (**A**) Experimental design of prime-and-trap protection studies. (**B**,**C**) Results of protection studies after challenge with 1 × 10^3^ WT purified Py spz administered four weeks after trapping with 2 × 10^4^ RAS + / − 7DW8-5 (**B**) or a dose de-escalation of RAS +/- 7DW8-5 (**C**). Protection data from N = 8–10 mice across two independent experiments (N = 5 from one experiment for the 5 × 10^2^ ID-RAS group in (**C**)). (**D**) Results of protection studies after challenge with 1 × 10^3^ WT purified Py spz administered four weeks after trapping with RAS + / − 7DW8-5 administered IV or ID ULV (ultra-low volume, 2.5 μL, X2 injections). Protection data from N = 10 mice across two independent experiments (N = 5 from one experiment for no adjuvant and 5 × 10^2^ ID + 7DW8-5 groups). Protection data was analyzed with Fisher Exact Test, ***p < 0.001, **p < 0.01, ns *p* > 0.05. (**E**) Naïve mice were immunized with cryo-RAS IV (100 μL) or ID ULV (2.5 μL, X2 injections) + / − 7DW8-5. Four hours post injection livers (left), and popliteal draining lymph nodes (PO dLN) (right) were excised and processed for real-time reverse transcription polymerase chain reaction (RT-PCR) to measure parasite burden with18S pan *Plasmodium* primers. Error bars represent the SD of the mean of N = 10 mice from two experiments. PO dLN samples were collected from the injected side and samples were processed in two pools of N = 5 alike dLN (one pool for IV group). (**F**) Naïve mice were challenged with 1 × 10^3^ infectious Py spz IV (100 μL), ID ULV (2.5 μL, X2 injections) or ID STV (standard volume,10 μL, X2 injections). 44 h post Py challenge livers were excised and processed for RT-PCR to measure liver stage parasite burden with 18S pan *Plasmodium* primers. Error bars represent the SD of the mean of N = 8–14 mice across two independent experiments (N = 3 mice for Naïve group). RT-PCR data was analyzed with Kruskal–Wallis test with Dunn’s multiple comparisons, ***p < 0.001, **p < 0.01, ns *p* > 0.05. ND = Not Detected. RT-PCR data are shown as absolute 18S rRNA copy numbers based on absolute calibrators.

Consistent with previous reports^11^, we found that ggCSP prime-and-ID RAS trap using standard ID injection volumes (STV) did not protect BALB/cJ mice against Py spz challenge (Fig. 1A,B). Although 7DW8-5 appears to be dispensable for IV-RAS in prime-and-trap^20^, we hypothesized that the adjuvant could improve the efficacy of ID-RAS by helping to overcome the tolerogenic environment of the skin^25,30,50^. To investigate this, mice were trapped with 2 × 10^4^ ID-RAS + / − 7DW8-5 and then challenged four weeks later with 1 × 10^3^ IV-administered Py spz (IV-spz). We found that protection induced by ID-RAS was significantly improved from 10 to 50% by the addition of 7DW8-5 (Fig. 1B). Additionally, protection was further improved to 80% by decreasing the administration volume from 50 µL to 10 µL, which was not significantly different from the 100% protection achieved by IV-RAS trap (Fig. 1B). This suggested that ID-RAS trapping could be effective in prime-and-trap when combined with the potent adjuvant 7DW8-5. Next, we sought to determine if the ID-RAS dose could be de-escalated while maintaining high levels of sterile protection, as was observed for IV-RAS^20^. However, reducing the dose of ID-RAS to 5 × 10^3^ or 5 × 10^2^ in 10 µL completely abrogated protection, despite the presence of the adjuvant (Fig. 1C). Taken together, this data demonstrates that prime-and-ID-trap is significantly improved by 7DW8-5 and by decreasing the ID injection volume to 10 µL, but that these changes are insufficient to de-escalate the ID-RAS dose.

### Prime-and-ultra-low volume 7DW8-5-adjuvanted ID-RAS trap completely protects mice against Py spz challenge

Previous studies found that fewer ID-RAS home to the liver compared to IV-RAS and suggest this as a primary reason why ID-RAS was less effective^28,29^. We hypothesized that differential parasite liver burdens after RAS administration could be responsible for the difference in protection observed when trapping with ID-RAS in 50 µL versus 10 µL. Moreover, since spz are known to migrate out of the skin in a process that requires surface contact^33^, we reasoned that by further reducing the volume used for ID-RAS, we could improve the motility of the spz to allow them to more effectively migrate out of the skin and home to the liver. To investigate the impact of injection volume on ID-spz liver burden, we co-administered de-escalating doses of the ID-RAS + 7DW8-5 trap in ultra-low volumes (ULV) of 2.5 µL. We found that 100% of the mice trapped with 2 × 10^4^ ULV ID-RAS + 7DW8-5 were protected against spz challenge (Fig. 1D). Additionally, the dose of ID-RAS could be reduced four-fold to 5 × 10^3^ RAS with only a modest loss of protection. However, protection was completely lost and only few parasites were detected in the liver when the dose was reduced to 5 × 10^2^ RAS (Fig. 1D; Supplementary Fig. 3). This suggests that the number of ID-RAS required to achieve protection in our model is between 5 × 10^2^ and 5 × 10^3^ spz and that prime-and-ULV ID-RAS + 7DW8-5 trap vaccination is equivalently protective at four weeks to our previously established prime-and-IV-RAS trap strategy^20^.

To confirm that 7DW8-5 was not detrimental to spz viability, we examined if the co-administration of ULV ID-RAS and 7DW8-5 impacted the number of spz that reached the liver. Previous ID-spz studies demonstrated that ID-spz travel to the liver via lymphatic and vascular systems, with a significant portion detectable in the draining lymph node^51^. To investigate these relevant tissue sites, naïve mice were immunized with 2 × 10^4^ ULV ID-RAS + / − 7DW8-5. Four hours later, livers and the ipsilateral popliteal draining lymph nodes (PO dLN) were harvested to quantify parasite liver burden by RT-PCR. The parasite liver burden was found to be similar across all groups, but ULV ID-RAS groups had substantially higher parasite loads in the PO dLN compared to IV-RAS (Fig. 1E). This data suggests that 7DW8-5 does not impact spz homing or liver invasion and that equivalent high numbers of parasites invade the liver following 2 × 10^4^ IV-RAS or ULV ID-RAS. Next, we compared the parasite liver burden following IV- or ID- spz challenge in a STV or ULV. We found that both IV-spz and ULV ID-spz yielded similar numbers of parasites in the liver, but STV ID-spz parasite load was significantly lower (Fig. 1F). Together, this data validates that ID-spz utilize lymphatics and vascular systems to home to the liver and that when injected in an ULV, ID-RAS reach the liver in equivalent numbers as IV-RAS.

Finally, we asked whether active spz motility in the skin and during liver invasion was critical for protection for ULV ID-RAS + 7DW8-5. Non-motile, heat-killed spz (HK-spz) cannot actively migrate, do not invade hepatocytes, and do not achieve sterile protection against IV-spz challenge in mice^20,52^. Similarly, here we found that mice trapped with IV- or ID- HK-spz + / − 7DW8-5 did not provide significant protection against spz challenge (Supplementary Fig. 4). This data confirms the critical importance of spz motility for prime-and-trap vaccination.

### 7DW8-5 potentiates ultra-low volume repeated ID-RAS only vaccination

RAS-only vaccines administered by direct venous inoculation 3–5 times are a benchmark experimental malaria vaccination strategy that achieves sterile protection in mice and humans (reviewed in^5,53^). Thus, we investigated if ULV ID-RAS was compatible with repeated RAS-only vaccination. To assess this, mice were immunized with 2 × 10^4^ ULV ID-RAS + / − 7DW8-5 three times at one-month intervals. Repeated IV-RAS routinely achieves 100% sterile protection in the BALB/cJ mouse model and was used as the benchmark in this experiment^53^. Here repeated dosing of ULV ID-RAS + 7DW8-5 was as protective as repeated IV-RAS (Supplementary Fig. 5). Thus, using the same spz dose, ID-RAS + 7DW8-5 is as equivalently protective as IV-RAS in both prime-and-trap and repeated RAS-only vaccination strategies.

### Prime-and-ULV 7DW8-5 adjuvanted ID-RAS trap induces CSP-specific liver CD8^+^ T cells

**Figure 2:**
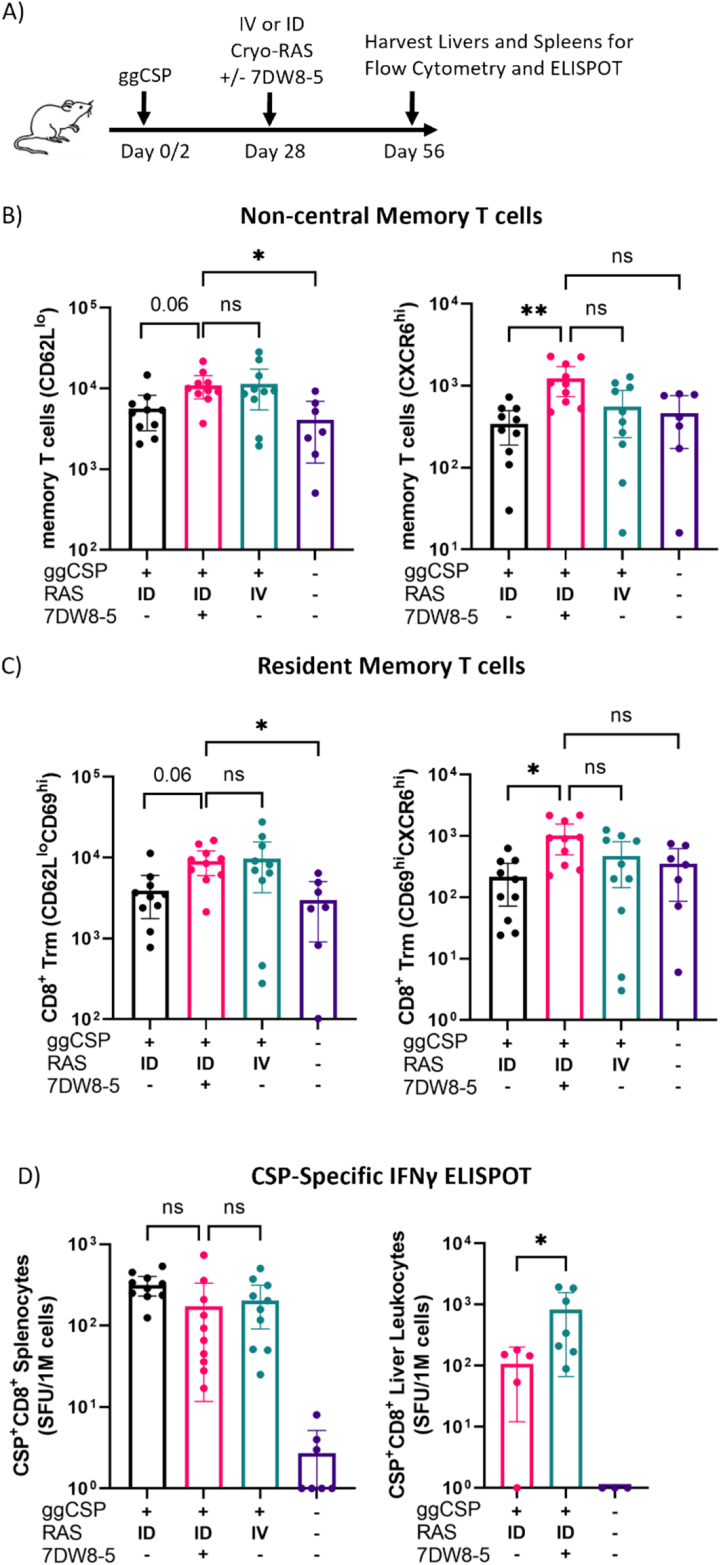
Prime-and-ULV 7DW8-5 adjuvanted ID-RAS trap induces CSP-specific liver CD8^+^ T cells. (**A**) Experimental design of prime-and-trap studies. (**B**) Flow cytometry of CD44^hi^/CD62L^lo^ (left) or CD44^hi^/CD62L^lo^/CXCR6^hi^ (right) CD8^+^ liver memory T cells from (**A**) livers. (**C**) Flow cytometry of CD44^hi^/CD62L^lo^/CD69^hi^ (left) or CD44^hi^/CD62L^lo^/CD69^hi^/CXCR6^hi^ (right) CD8^+^ liver Trm cells from (**A**) livers. (**D**) IFNγ ELISPOT from (**A**) 5 × 10^5^ splenocytes (left) or 3 × 10^5^ liver leukocytes (right) stimulated with CSP peptide (SYVPSAEQI) or DMSO vehicle control. Data was normalized to vehicle control. Error bars represent 95% confidence interval of the mean from N = 7–10 mice across two experiments (N = 5–7 for Liver ELISPOT). Data was analyzed with Kruskal–Wallis test with Dunn’s multiple comparisons, **p < 0.01, *p < 0.05, ns *p* > 0.05. All ULV ID-RAS injections were 2.5 μL, X2 injections.

Next, we investigated the magnitude of the liver CD8^+^ T cell responses induced by ID-RAS + 7DW8-5. Mice were trapped with IV-RAS or ULV ID-RAS + 7DW8-5 as before, and four weeks post-trapping, spleens and livers were harvested for CD8^+^ T cell analysis (Fig. 2A). CD8^+^ Trm cells in the liver were defined as either CD69^hi^/KLRG1^lo^ or CD69^hi^/CXCR6^hi^ as previously described^18,19^. We found that overall, the number of CD44^hi^/CD62L^lo^ non-central memory CD8^+^ T cells were reduced in the ID-RAS group compared to the ID-RAS + 7DW8-5 group (Fig. 2B, Supplementary Fig. 6). Additionally, we found a similar trend in the CD69^hi^/CXCR6^hi^ Trm cells (Fig. 2C); however, the CD69^hi^/KLRG1^lo^ Trm cells were similar in all the immunized groups (Supplementary Fig. 6). Since equivalently high numbers of CD69^hi^/KLRG1^lo^ Trm cells were observed for treatments that differed in protection outcomes in the challenge experiments above, the data suggests that CD69^hi^/KLRG1^lo^ Trm cells defined by phenotypic surface markers alone may be insufficient to explain protection. Consistent with other malaria vaccination studies in rodents, in our model CD69^hi^/CXCR6^hi^ defined Trm cells may be especially critical for protection^18,54^. We further characterized the CSP-specific CD8^+^ T cell responses in the spleen and liver via ELISPOT. Consistent with previous reports, we found that compared to ID-RAS groups, ID-RAS + 7DW8-5 induced significantly more CSP-specific responses in the liver, but not the spleen (Fig. 2D)^27^. Taken together, our findings corroborate previous work suggesting liver CSP-specific CD8^+^ T cells are induced by RAS vaccination and are likely the most important immune cell populations for protection in mice.

### Prime-and-7DW8-5 adjuvanted ID-RAS trap induces inflammatory innate immune responses in the liver

**Figure 3:**
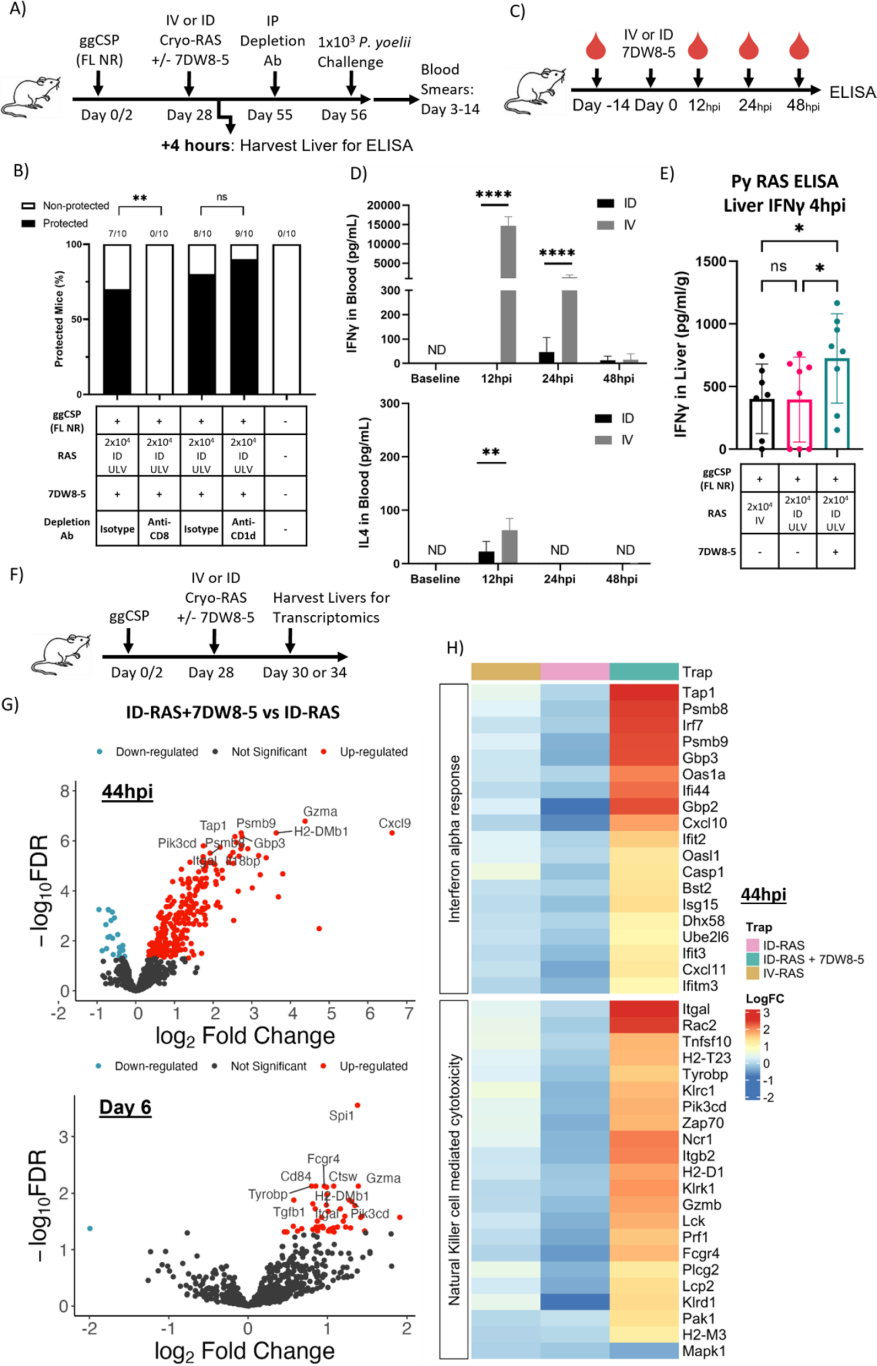
Prime-and-7DW8-5 adjuvanted ID-RAS trap induces inflammatory innate immune responses in the liver. (**A**) Experimental design of prime-and-trap studies in (**B**,**E**). (**B**) Results of protection studies after challenge with 1 × 10^3^ WT purified Py spz administered four weeks after trapping with RAS + / − 7DW8-5 administered ID ULV (2.5 μL, X2 injections). Depletion antibodies were injected IP into all animals 24 h before challenge as indicated. Protection data from N = 10 mice across two independent experiments and analyzed with Fisher Exact Test, **p < 0.01, ns *p* > 0.05. (**C**) Experimental design of blood plasma ELISA studies. (**D**) Cytokine levels, IFNγ (top) and IL4 (bottom), in mouse blood plasma following IV (100 μL) or ID (10 μL, X2 injections) administration of 7DW8-5. IV data reproduced from Watson et al.^20^ for comparison. Error bars represent the SD of the mean of N = 10 mice across two independent experiments. ELISA data analyzed with Mann–Whitney Tests, ****p < 0.0001, **p < 0.01. (**E**) Four hours after trapping with cryo-RAS IV (100 μL) or ID ULV (2.5 μL, X2 injections) with or without 7DW8-5 livers were excised and processed for ELISA to measure IFNγ. Error bars represent SD of N = 7–8 mice across two experiments. Data was analyzed with Kruskal–Wallis test with Dunn’s multiple comparisons, **p < 0.01, *p < 0.05, ns *p* > 0.05. (**F**) Experimental design of transcriptomics studies after trapping with RAS + / − 7DW8-5 administered IV (100 μL) or ID (10 μL, X2 injections). Transcriptomic data in G-H represents data from N = 3 mice per group from one experiment for each timepoint. Group averages are displayed for all groups. (**G**) Volcano plot of differentially expressed genes comparing ID-RAS versus ID-RAS + 7DW8-5 from (**F**) livers harvested at 44 h post-injection (hpi) (top) or Day 6 post injection (bottom). Genes in red have higher expression in the ID-RAS + 7DW8-5 group and genes in blue have higher expression in the ID-RAS group. Top 10 most significant genes are labeled. (**H**) Heatmap and hierarchal clustering of genes from livers harvested at 44hpi that contain at least one significant differentially expressed gene within the selected MSigDB hallmark interferon alpha pathway and KEGG natural killer cell mediated cytotoxicity pathway. Data are represented as logFC as compared to control group only receiving ggCSP (no RAS). Significance defined as FDR Adj. *p* ≤ 0.05 and log2fold change of ± 1.

CD8^+^ T cells but not *i*NKT cells are critical for protection from spz challenge following RAS vaccination in mice^11,55^. However, the immunostimulatory mechanism by which 7DW8-5 acts is through binding CD1d-expressing APCs and activating *i*NKT cells, so we investigated if *i*NKT cells at the time of challenge were required for protection^56^. We depleted or blocked CD8 or CD1d before challenge and found that protection was completely lost when CD8^+^ cells were depleted but was not impacted by the significant reduction of CD1d cells (Fig. 3A,B, Supplementary Fig. 7). Thus, we confirmed that prime-and-ID trap protection is likely driven primarily by CD8^+^ cells.

Previous studies have shown that IV-administered 7DW8-5 induced a potent and transient spike of systemic IFN-γ (and to a lesser extent IL-4) in mouse blood^20^, but intramuscular (IM) administration of 7DW8-5 did not^57^. Consistent with this data, we found that ID administration of 7DW8-5 did not induce systemic IFN-γ or IL-4 (Fig. 3C,D). However, liver IFN-γ concentrations were significantly increased after prime-and-ULV ID-RAS + 7DW8-5 compared to the unadjuvanted IV-RAS or ULV ID-RAS controls (Fig. 3E). This finding suggests that although ID-7DW8-5 does not induce systemic cytokine expression, it likely impacts local tissue cytokine expression.

Next, we explored the key factors in the liver responsible for the differential protection outcomes. We hypothesized that 7DW8-5 influences the innate immune responses in the liver, which subsequently influences the quality and polyfunctionality of the induced CD8^+^ memory T cell responses. To evaluate this, livers were harvested from vaccinated animals at 44 h and Day 6 post-trapping to explore gene expression changes induced by 7DW8-5 in the liver. Overall, unadjuvanted RAS immunization (IV-RAS or ULV ID-RAS) was the least immunogenic and showed few differentially expressed genes compared to the ggCSP only control animals (Supplementary Fig. 8). However, in the 44 h ULV ID-RAS + 7DW8-5 group, we found 119 and 154 differentially-expressed genes (FDR Adj. *p* ≤ 0.05 and log2 fold change of ± 1) compared to ggCSP only and ULV ID-RAS groups respectively (Fig. 3F,G). Most notably, genes associated with interferon signaling (IFNα and IFNγ responses), natural killer cytotoxicity, and antigen processing were significantly upregulated in the 7DW8-5 groups (Fig. 3H). Parasite liver burden was also measured at 44 h and showed a significant decrease of *Plasmodium* 18S rRNA copies in the 7DW8-5-adjuvated ULV ID-RAS group compared to IV-RAS (Supplementary Fig. 8). This data suggests that the kinetics of parasite clearance in the liver differ between ULV ID-RAS + 7DW8-5 and IV-RAS, which we propose is driven by the inflammatory effects of the adjuvant. In the Day 6 ULV ID-RAS + 7DW8-5 group, only 23 and 30 genes were differentially-expressed (FDR Adj. *p* ≤ 0.05 and log2 fold change of ± 1) compared to ggCSP only and ULV ID-RAS groups, respectively (Fig. 3F,G). At this later timepoint, genes associated with antigen processing and IFNγ responses were significantly upregulated in the 7DW8-5 groups. Taken together, this data indicates that co-administration of ID RAS + 7DW8-5 drives the immune environment in the liver toward a pro-inflammatory state that may be more favorable for CD8^+^ T cell memory formation.

### PyCSP antibodies induced by priming against non-repeat regions are not detrimental to ID-RAS trapping

All experiments thus far used the well-characterized and immunogenic CSP epitope (SYVPSAEQI, presented on H2-K^d^ MHC) for ggCSP priming, but this vaccine does not induce anti-CSP IgG antibodies​ (Supplementary Fig. 9). Vaccination with full length CSP protein is important for increasing epitope diversity and will likely be required for translation of the prime-and-trap vaccine strategy. However, it was not yet clear if antibodies induced by full-length CSP priming would be detrimental to ID-RAS trap since anti-spz antibodies are known to be active in the dermis^58^. The major repeat region of CSP binds the majority of potent spz neutralizing antibodies^59^, so we first cloned the full-length CSP gene—without the major repeat region—into our plasmid backbone (ggCSP full-length no repeat (FL NR)) (Supplementary Fig. 1). The intention of this construct was to maximize the antigenic landscape while eliminating the target of the most potent spz neutralizing antibodies. To evaluate antibody responses to priming, we compared anti-CSP antibodies induced by ggCSP (epitope), ggCSP (FL NR), or the pUb.3 plasmid backbone without the CSP insert (control DNA) via ELISA. As expected, only the mice immunized with ggCSP (FL NR) produced anti-CSP antibodies on day 28 (Supplementary Fig. 9), which due to the design of the ggCSP (FL NR) construct could be attributed to epitopes outside the repeat region.

**Figure 4:**
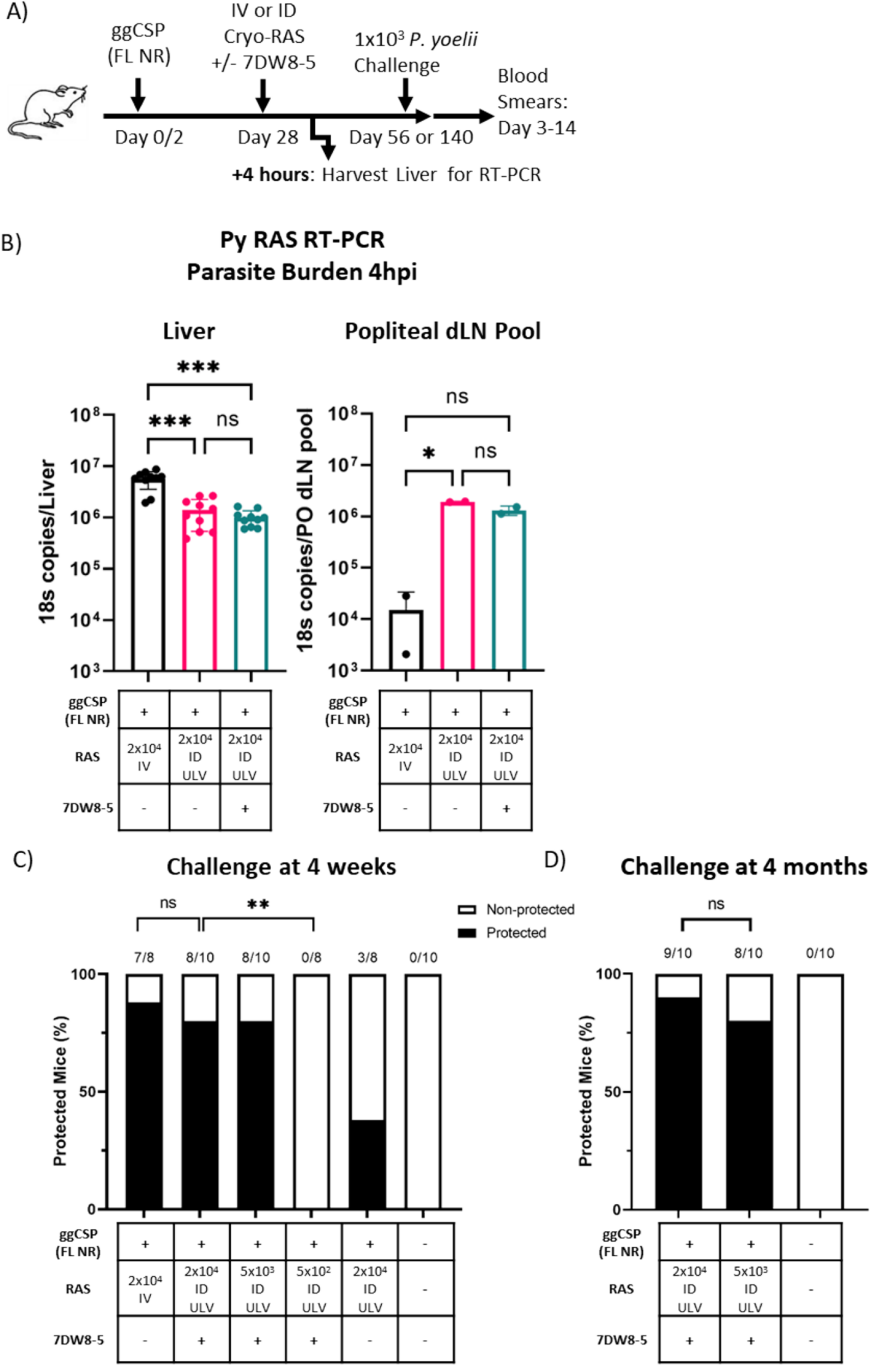
Antibodies to the non-repeat region of PyCSP do not affect the ID-RAS trapping. (**A**) Experimental design of prime-and-trap studies. (**B**) Four hours after trapping with RAS IV (100 μL) or ID ULV (2.5 μL, X2 injections) + / − 7DW8-5 livers (left) and popliteal draining lymph nodes (PO dLN) (right) were excised and processed for RT-PCR to measure parasite burden with 18S pan *Plasmodium* primers. RT-PCR data are shown as absolute 18S rRNA copy numbers based on absolute calibrator. Error bars represent SD of mean of N = 10 mice across two independent experiments. PO dLN samples were collected from the injected side and samples were processed in two pools of N = 5 alike dLN. RT-PCR data was analyzed with Kruskal–Wallis test with Dunn’s multiple comparisons, ***p < 0.001, *p < 0.05, ns *p* > 0.05. (**C**,**D**) Results of protection studies after challenge with 1 × 10^3^ WT purified Py spz administered four weeks (**C**) or four months (**D)** after trapping with RAS + / − 7DW8-5 administered IV or ID ULV (2.5 μL, X2 injections). Protection data from N = 8–10 mice across two independent experiments and was analyzed with Fisher Exact Test, **p < 0.01, ns *p* > 0.05.

Next, we investigated if these priming-induced antibodies targeting epitopes outside the major repeat region could impact the number of ID-RAS that reached the liver. We harvested livers and PO dLNs from mice primed mice with ggCSP (FL NR) and trapped with ULV ID-RAS + / − 7DW8-5 to compare the parasite burdens and evaluate spz exit from the skin. Although parasite liver burden was significantly reduced in the ULV ID-RAS trap groups compared to IV-RAS group, the levels were still high (Fig. 4A,B). This data suggests that the priming did indeed induce antibodies against the non-repeat regions of CSP that could impact ID-RAS homing to the liver, but that this impact was relatively minor. We hypothesized that the minor reduction in liver burden would not impact protection. Indeed, we found that similarly high levels of protection were achieved in ggCSP (FL NR) primed animals as observed in the ggCSP (epitope) primed mice despite trapping in the presence of anti-CSP antibodies and reduced liver burdens (Fig. 4C). Importantly, the trapping dose could still be reduced four-fold without a significant loss of protection (Fig. 4C). To evaluate the durability of protection, mice were similarly immunized, and protection was assessed four months post trapping. Strikingly, all mice were equivalently highly protected from spz challenge in both the high (2 × 10^4^) and low (5 × 10^3^) dose ULV ID-RAS + 7DW8-5 groups (Fig. 4D). This data demonstrated that antibodies against the non-repeat regions of CSP induced by priming with ggCSP (FL NR) were not detrimental to IV-RAS or ULV ID-RAS + / − 7DW8-5 trapping.

### High titers of exogenously-administered anti-CSP repeat region spz neutralizing mAb inhibit prime-and-trap vaccination

**Figure 5:**
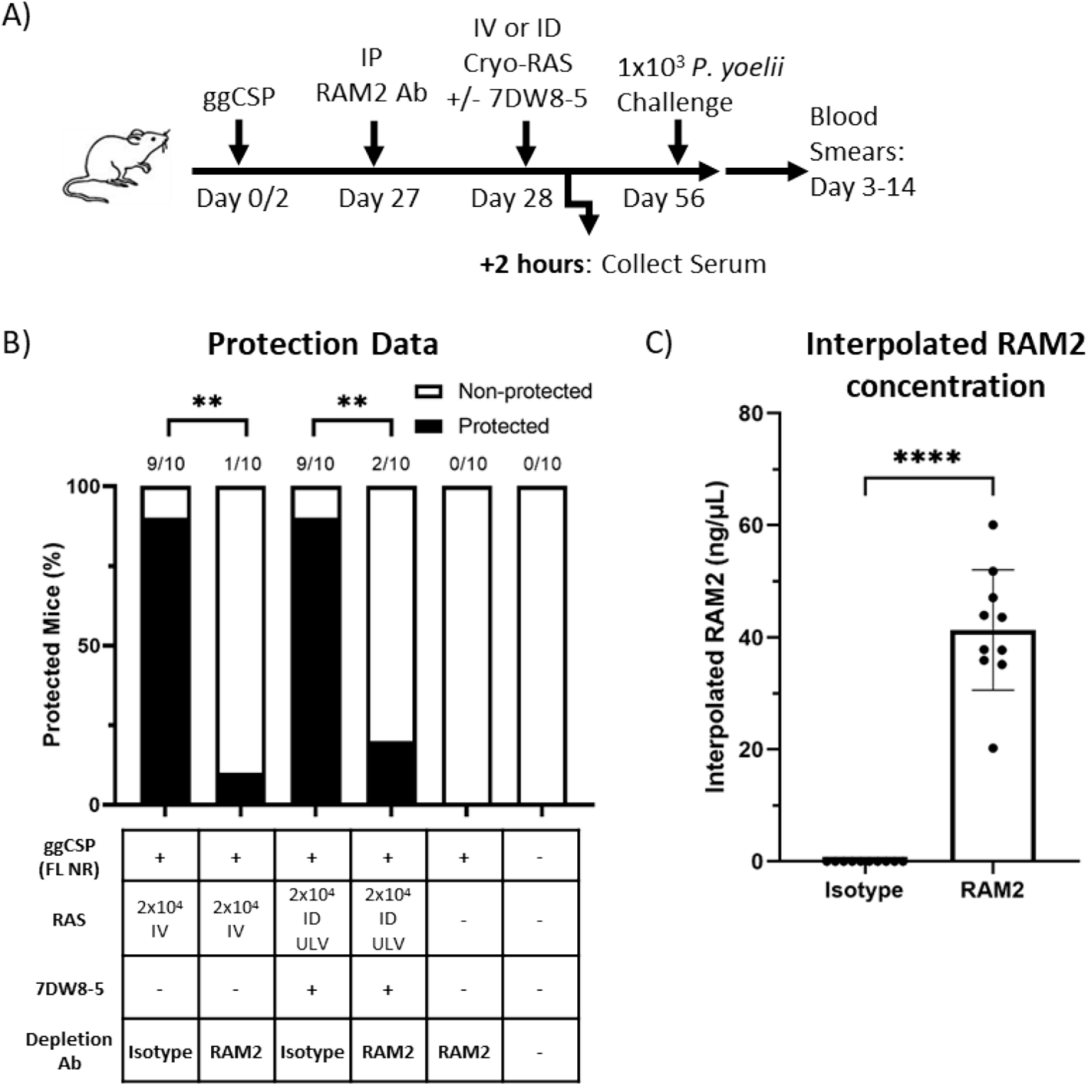
High titers of exogenously-administered spz neutralizing mAb inhibit prime-and-trap vaccination. (**A**) Experimental design of prime-and-trap studies. (**B**) Results of protection studies after challenge with 1 × 10^3^ WT purified Py spz administered four weeks after trapping with RAS + / − 7DW8-5 administered IV or ID ULV (2.5 μL, X2 injections). RAM2 or isotype control mAb was injected IP into mice 24 h prior to trapping as indicated. Protection data from N = 10 mice across two independent experiments and analyzed with Fisher Exact Test, **p < 0.01. (**C**) Results of anti-RAM2 serum ELISA from a subset of the (**B**) mice that received RAM2 or Isotype control. Error bars represent the SD of the mean of N = 10 mice from two experiments. ELISA data was analyzed with Mann–Whitney Test, ****p < 0.0001.

Attenuated spz vaccines are more effective in malaria-naïve individuals (reviewed in^3^), which may in part be due to pre-existing antibodies in malaria-experienced individuals neutralizing vaccine spz before they can reach the liver. Thus, we sought to evaluate a scenario where high titers of pre-existing anti-spz antibodies were present prior to prime-and-trap vaccination. The major repeat region of CSP is the target of the most potent spz neutralizing antibodies^59^, and these antibodies can be found in varying concentrations in naturally-exposed individuals^60,61^. We therefore investigated if prime-and-trap would still be effective if RAS were administered in the presence of high titers of potent pre-existing spz neutralizing antibodies. RAM2 is a spz neutralizing monoclonal antibody (mAb) that binds PyCSP with high affinity and induces high rates of sterile protection against mosquito bite challenge in mice^41^. Here, we examined the impact of immunizing in the presence of high titers of RAM2. Mice were primed with ggCSP (FL NR) and trapped with 2 × 10^4^ IV-RAS or ULV ID-RAS + 7DW8-5 (Fig. 5A). Importantly, 24 h prior to trapping, 150 µg RAM2 or matched isotype control mAb were administered. Protection induced by prime-and-trap was completely abrogated by the presence of high titers of RAM2 antibodies regardless of whether the RAS trap was delivered by IV or ULV ID (Fig. 5B). Circulating anti-CSP mAb titers were confirmed to be ~ 40 ng/µL at the time of immunization by ELISA (Fig. 5C). To further elucidate the impact of RAM2 on vaccine spz, we measured the parasite liver burden and found that RAM2 did not significantly reduce *Plasmodium* 18S rRNA copies in the liver of the IV-RAS group, but significantly reduced the liver burden of the ULV ID-RAS group (Supplementary Fig. 9). Taken together, this data suggests that high titers of spz invasion blocking antibodies may interfere with prime-and-trap or attenuated spz vaccine efficacy, but notably, sterile protection was similarly impacted in both IV- and ULV ID trapping groups.

### Single dose vaccination

The final set of experiments sought to investigate if a condensed single day prime-and-trap schedule could be effective. Another group reported that a single day prime-and-trap vaccine induced sterile protection from *P. berghei* spz challenge in C57BL/6 mice^18,62^. An accelerated vaccination regimen would further enhance and simplify the prime-and-trap vaccine. However, previously a condensed single day prime-and-trap (ggCSP and IV-RAS administered on the same day) induced significantly less CSP-specific CD8^+^ Trm cells in the liver compared to the standard prime-and-trap (ggCSP and IV-RAS administered 4 weeks apart)^19^. Thus, the final experiments aimed to investigate if the addition of 7DW8-5 to the condensed single day prime-and-trap would improve the vaccine efficacy. Interestingly, IV-RAS + 7DW8-5 administered on a single day with or without ggCSP priming was protective in mice (Supplementary Fig. 10). On the contrary, ULV ID-RAS + 7DW8-5 administered on a single day with or without ggCSP priming was not protective in mice (Supplementary Fig. 10). This data suggests that the mechanism of protection induced by IV- or ID-RAS + 7DW8-5 may differ, and future studies are warranted to uncover the correlates of protection. In summary, a single immunization of IV-RAS + 7DW8-5 (but not ID-RAS + 7DW8-5) is an effective and simple vaccine strategy in mice and has significant translational potential into non-human primates and humans.

## Discussion

Malaria cases increased by ~ 12% in 2020^1^, highlighting the importance of a more effective vaccine that can prevent clinical manifestations and stop further transmission. Decades of pre-clinical and clinical studies of RAS vaccines have demonstrated the safety, feasibility, and efficacy of this vaccine strategy^4,8,9,11^, but efforts to simplify and improve administration may further improve the impact of spz vaccines. ID vaccine administration is of growing interest due the increased immunogenicity and dose sparing potential^23^. A systematic review and meta-analysis found that ID immunization is dose-sparing for many non-malaria infectious diseases as compared to IM or subcutaneous (SC) administration (reviewed in^23^). However, IV administration of RAS is much more efficient than IM or SC administration and ID-RAS vaccination has previously required ~ 7X *higher* doses to reach equivalent protection as IV-RAS^11^. Here, we explored two methods to increase the efficacy of prime-and-ID-RAS vaccination: 1) reduction in the administration injection volume, and 2) use of a glycolipid adjuvant. We demonstrate that prime-and-trap with an equivalent dose of ID-RAS is as effective as IV-RAS when co-administered in an ultra-low volume with the glycolipid adjuvant 7DW8-5. Thus, both microvolumes and adjuvanting were critical for the success of ID-RAS trap vaccination.

In human and mouse studies, ID-RAS vaccine failures were attributed to regulatory cellular responses^25^ and low parasite burdens in the liver^28,29^. Our studies aimed to further explore these hypotheses. First, we found that ggCSP priming followed by co-administration of ID-RAS + 7DW8-5 significantly improved protection from spz challenge. Glycolipid adjuvants, including 7DW8-5, bind CD1d expressing APCs and are known to induce a cascade of immune cell activation^35^. In our model, the 7DW8-5 adjuvant effects appeared to be necessary to modulate a favorable pro-inflammatory immune environment in the liver. Significant levels of protection were never achieved in our hands after ID-RAS immunization without 7DW8-5. This finding is supported by the previous literature also showing that modulation of the immune environment with adjuvants or epidermal disruption improves non-IV RAS administration^50,63,64^, and that adjuvants or other pro-inflammatory modulating factors are likely required to overcome tolerogenic skin responses and/or regulatory liver responses for efficacious ID-RAS vaccination^25,30^. Second, we found that protection achieved from prime-and-ID-RAS + 7DW8-5 trap vaccination could be further improved by reducing the ID injection volume. Others have also noted that lower volumes may improve the migration capacity of spz out of the dermis^29,65^. In line with prior work, we found that ULV ID-RAS significantly increased the number of parasites that reached the liver compared to STV ID-RAS. However, achieving parasite liver burdens equivalent to IV dosing was not sufficient for protection. Thus, our data supports the hypothesis that a combination of high parasite liver burden and a pro-inflammatory liver immune environment is required for efficacious ID-RAS vaccination.

Inducing high levels of malaria-specific CD8^+^ Trm cells in the liver is required for RAS vaccine efficacy^18^. We found higher numbers of CSP-specific CD8^+^ T cells in the livers of ID-RAS + 7DW8-5 mice compared to ID-RAS. Consistent with other studies, we also found that CXCR6^hi^-defined CD8^+^ Trm cells were important for sterile protection in our model^18,27^ . CXCR6 has been implicated as a key liver homing marker that may be critical for memory T cell maintenance in the liver^54^. In addition, our Nanostring analysis found that *Cxcr3* and *Cxcr6* were upregulated in the protected groups (ID-RAS + 7DW8-5 and IV-RAS) compared to the unprotected controls (ggCSP and ID-RAS) (Supplementary Fig. 8 and Supplementary Files 1, 2). It is tempting to speculate that the quality and functionality of the CD8^+^ Trm cells is driving the protective differences, but the data cannot definitively address this question at this time. In addition to the number of CD8^+^ Trm cells shown here, our findings warrant future exploration into polyfunctionality of vaccine-induced CD8^+^ Trm cells in the liver.

Further evidence for the important role of CD8^+^ T cells in conferring protection after prime-and-trap vaccination came from the antibody depletion experiments. At the time of challenge, CD8^+^ cells but not CD1d-expressing cells, were critical for sterile protection. While a potential limitation of our study is that we did not achieve full CD1d cell neutralization, the data nonetheless agrees with several other studies in CD1d knockout mice that also concluded CD1d was dispensable at the time of challenge for RAS vaccine efficacy^18,66^. We propose that CD1d-expressing cells are critical for ID-RAS + 7DW8-5 trapping to bind 7DW8-5 and induce a strong pro-inflammatory immune response to activate and form CD8^+^ T cells in the liver. Then, if induced correctly, liver CD8^+^ T cells may be sufficient for protection from spz challenge. In this model, we propose that CD1d-expressing cells are needed at the time of vaccination, but are dispensable for sensing parasites or activating CD8^+^ T cells at the time of challenge.

Given the clear importance of CD8^+^ T cells for conferring protection, we also investigated the events during vaccination to that gave rise to either protective or non-protective responses. Innate immune responses during vaccination are known to be critically important for shaping the subsequent adaptive response, including the quality and the durability of CD8^+^ T cell responses^66–68^. Our targeted gene expression studies using the Nanostring platform provided helpful insight into the immune response in the liver after trapping. These studies revealed several key findings. First, despite the high RAS dose used for immunization, commercially-produced aseptic, cryo-RAS are highly purified and did not induce innate inflammatory responses in the liver regardless of administration route. Second, the addition of 7DW8-5 completely altered the innate response to trapping in the liver, with interferon signaling and other pro-inflammatory associated pathways significantly upregulated in prime-and-ULV ID-RAS + 7DW8-5 vaccinated mice in comparison to unadjuvanted groups. Based on these data, we speculate that interferon signaling and pro-inflammatory responses at the time of trapping likely result in a recruitment of leukocytes, an increase in antigen processing and presentation, and enhanced memory CD8^+^ Trm cell formation in the liver. We also note that other groups have studied adaptive regulatory cellular responses to ID-RAS and detected higher CD4^+^ regulatory immune responses and lower CD8^+^ T cell activation seven days post-spz administration^25^. Our gene expression analysis at an equivalent timepoint (Day 6) did not reveal significant differences in adaptive regulatory cellular response pathways. Future studies could phenotype the liver T cells at this timepoint to further characterize adaptive regulatory cellular responses including T-cell exhaustion to address whether 7DW8-5 overcomes the previously noted adaptive regulatory responses. Such studies would enable a more complete picture of the full suite of events leading to protective immune responses to prime-and-trap vaccination in the liver.

Antibodies also play an important role in pre-erythrocytic vaccine protection. Previous studies suggested that the majority of antibodies act to inhibit spz in the skin^58^, but increasingly the importance of anti-spz antibodies in mediating clearance of parasites outside of the skin are appreciated^69^. We hypothesized that ID-RAS vaccines would be inhibited to a greater extent by anti-spz antibodies compared to IV-RAS, and we found that this was indeed the case. Using ggCSP (FL NR) priming, the liver burden of ID-RAS (but not IV-RAS) was significantly reduced by the anti-CSP antibodies induced by priming, but protection was unaffected. However, regardless of the administration route, protection was significantly impacted by the presence of high titers of potent anti-CSP repeat region mAb exogenously administered prior to RAS trapping. This observation was unexpected as we hypothesized that protection induced from ID-RAS would be more impacted by high titer mAb than IV-RAS. Thus, more studies are warranted to understand the impact of anti-spz antibody responses to both prime-and-IV trap and IV-RAS only vaccines. Such studies could provide important information about the levels of circulating pre-existing antibodies that inhibit successful spz vaccination.

Finally, one of the key findings here is that administration in an extremely low volume is critically important for successful ID-RAS vaccination. We expect that these ultra-low volumes will still be necessary when scaling up ID-RAS to larger animal models or humans. Inoculation in ultra-low volumes improves spz motility in the skin and allows spz to efficiently invade blood vessels and lymph to home to the liver and dLN, respectively^34^. In our report, 2.5 µL was selected as the smallest volume that could be reliably prepared in the research laboratory for pre-clinical mouse injections. This volume is very low compared to standard ID-administered vaccines (50–100 µL), but still higher than the estimated mosquito saliva injection of < 1 µL. Additionally reducing the injection volume to more closely mimic the volumes delivered during mosquito probing may further improve ID-RAS. Studies with PfSPZ Vaccine and PfSPZ Challenge have shown that direct venous inoculation of 0.3–0.5 mL of PfSPZ through a 25-gauge needle is extremely well tolerated, simple, and reliable when administered by personnel after minimal training^70^. Conceptually, ID administration appears easier, but reproducibly injecting even 50–100 µL ID at an accurate depth and volume with a standard single-needle syringe can be challenging^71^. However, accurate and reliable ID injection may be possible through the development of a microarray needle patch or another as-yet-to-be-developed administration device. Thus, ID engineering innovations could revolutionize ID-RAS administration in the field and allow simple, quick, and pain-free administration of ULV ID-RAS.

In summary, the use of ultra-low volumes for ID-RAS administration significantly improves the number of vaccine parasites that home to and invade the liver. However, in the context of prime-and-trap vaccination, the combination both 7DW8-5 and ULV ID-RAS at the trapping step is required for complete protection from spz challenge. Taken together, prime-and-ULV ID-RAS + 7DW8-5 trap is a highly effective vaccine in mice that has significant translational potential. Combined with the recent report of in vitro production of *Plasmodium falciparum* sporozoites^72^, our insights about lower administration volumes and adjuvants provide a potential path forward for simplifying attenuated sporozoite vaccination.

### Supplementary Information

Supplementary Information 1.

Supplementary Information 2.

Supplementary Information 3.

## Data Availability

Gene expression data are available within the article and its supplementary data files. All data are available from the corresponding author upon request.
