# Supplementary material for: Ultra-low volume intradermal administration of radiation-attenuated sporozoites with the glycolipid adjuvant 7DW8-5 completely protects mice against malaria
Source: Sci Rep. 2024 Feb 4;14:2881. doi: 10.1038/s41598-024-53118-9 (PMC10838921; doi:10.1038/s41598-024-53118-9)
Supplement: Supplementary file 1 — Supplementary Information 1. [file 41598_2024_53118_MOESM1_ESM.pdf]

SUPPLEMENTARY FIGURES

Specificity	Clone	Fluorochrome	Vendor	Catalog
CD3e	145-2C11	BUV 395	BD	563565
B220	RA3-6B2	BV711	BioLegend	103255
CD4	GK1.5	Alexa Fluor 700	BioLegend	100429
CD8a	53-6.7	BV421	BD	563898
CD69	H1.2F3	BV510	BD	563030
CD44	IM7	Alexa Fluor 488	BioLegend	103015
CD62L	MEL-14	PE-Cy7	BD	560516
KLRG1	2F1	PerCP-Cy5.5	BioLegend	138418
CXCR6	221002	PE	Fisher Scientific	FAB2145P100
CSP-tetramer	-	APC	NIH	-
APC	-	Streptavidin-APC	Prozyme	PJ27S
Zombie NIR™ Fixable Viability	-	NIR	BioLegend	423105
Fc CD16/32	2.4G2	-	BD	553141
Counting Beads	-	-	Polyscience	18328-5

Supplementary Table 1: Flow cytometry materials

Flow cytometry reagents and antibodies that were used to assess liver $CD8^{+}$ cells.

A) **CSP FULL-LENGTH** **(289aa)**
MKKCTILVVASLLLVDSLLPGYGQNKSVQAQRNLNELCYNEENDNKLYHVLNSKNGKIYNRNIVNRLLGDALNGKPEEK
KDDPPKDGNKDDLPKEEKKDDLPKEEKKDDPPKDPKKDDPPKEAQNKLNQPVVADENVDQ | **GPGAPQGPGAPQG**
PGAPQGPGAPQEPPQQPPQQPPQQPPQQ | PRPQPDGNNNNNNNNGNNNED**SYVPSAEQI**LEFVKQISSQLTEE
WSQCSVTCGSGVRVRKRKNVNKQPENLTLEDIDTEICKMDKCSSIFNIVSNSLGFVILLVLVFFN

CSP FULL-LENGTH NO REPEAT (CSP FL NR) (248 aa)
MKKCTILVVASLLLVDSLLPGYGQNKSVQAQRNLNELCYNEENDNKLYHVLNSKNGKIYNRNIVNRLLGDALNGKPEEK
KDDPPKDGNKDDLPKEEKKDDLPKEEKKDDPPKDPKKDDPPKEAQNKLNQPVVADENVDQ | PRPQPDGNNNNNN
NNGNNNED**SYVPSAEQI**LEFVKQISSQLTEEWSQCSVTCGSGVRVRKRKNVNKQPENLTLEDIDTEICKMDKCSSIFNI
VSNSLGFVILLVLVFFN

CSP MINIGENE **(25 aa)**
NNNNNGNNNE**SYVPSAEQI**GLSERH

Major Repeat Region (41aa)
MHC I epitope (9aa)

B) **Agarose restriction digest gel**

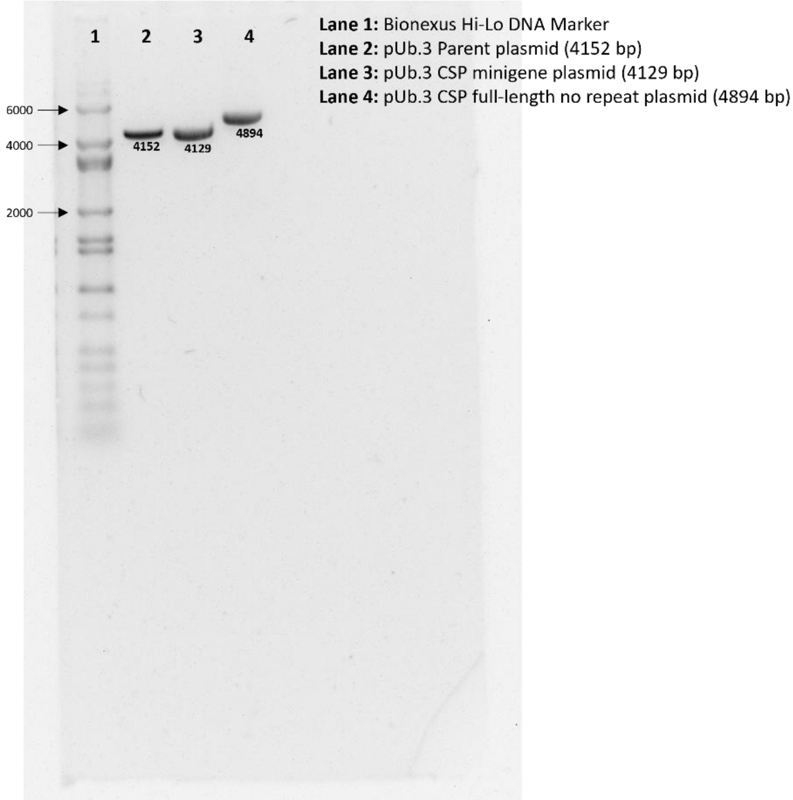

Lane 1: Bionexus Hi-Lo DNA Marker
Lane 2: pUb.3 Parent plasmid (4152 bp)
Lane 3: pUb.3 CSP minigene plasmid (4129 bp)
Lane 4: pUb.3 CSP full-length no repeat plasmid (4894 bp)

Supplementary Figure 1: CSP DNA vaccines

A) Py CSP DNA vaccine insert amino acid sequences.

B) Agarose restriction digest gel of 100 ng plasmid digested and cut once with a restriction enzyme to linearize to visualize true sizes on the gel. A Bionexus Hi-Lo DNA Marker ladder was run in lane 1 for reference with key band sizes listed in base pairs (bp).

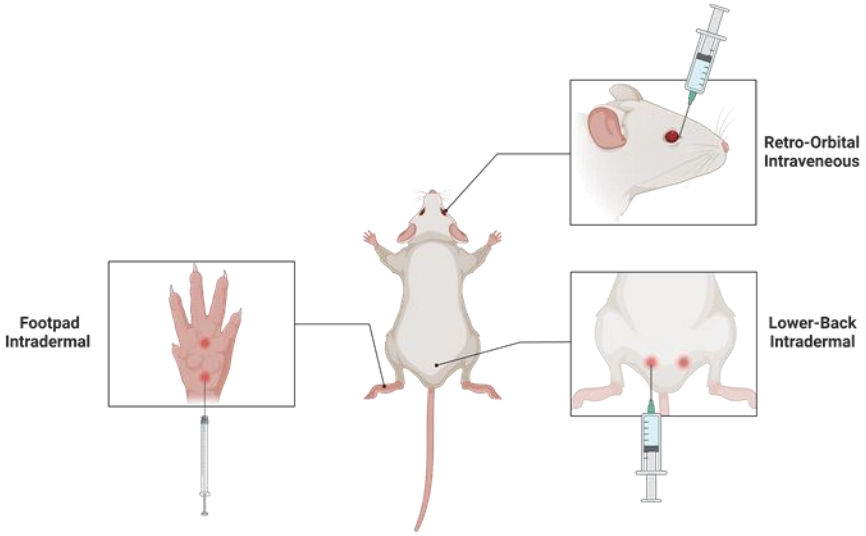

Supplementary Figure 2: Mouse injection sites

Images adapted from BioRender template.

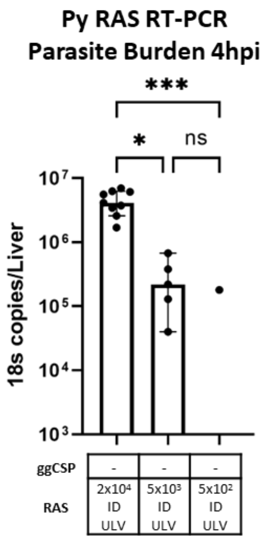

Supplementary Figure 3: Ultra-low volume ID-RAS dose de-escalation parasite liver burden

Naïve mice were immunized with cryo-RAS ID ULV (2.5 µL, X2 injections). Four hours post injection livers were excised and processed for real-time reverse transcription polymerase chain reaction (RT-PCR) to measure parasite burden with pan-*Plasmodium* 18S rRNA primers. Error bars represent the SD of the mean of N=5 mice from one experiment. RT-PCR data was analyzed with Kruskal-Wallis test with Dunn's multiple comparisons, ***p<0.001, *p<0.05, ns p>0.05. RT-PCR data are shown as absolute 18S rRNA copy numbers based on absolute calibrators. $2x10^4$ ID ULV data (left column) reproduced from Figure 1E for comparison.

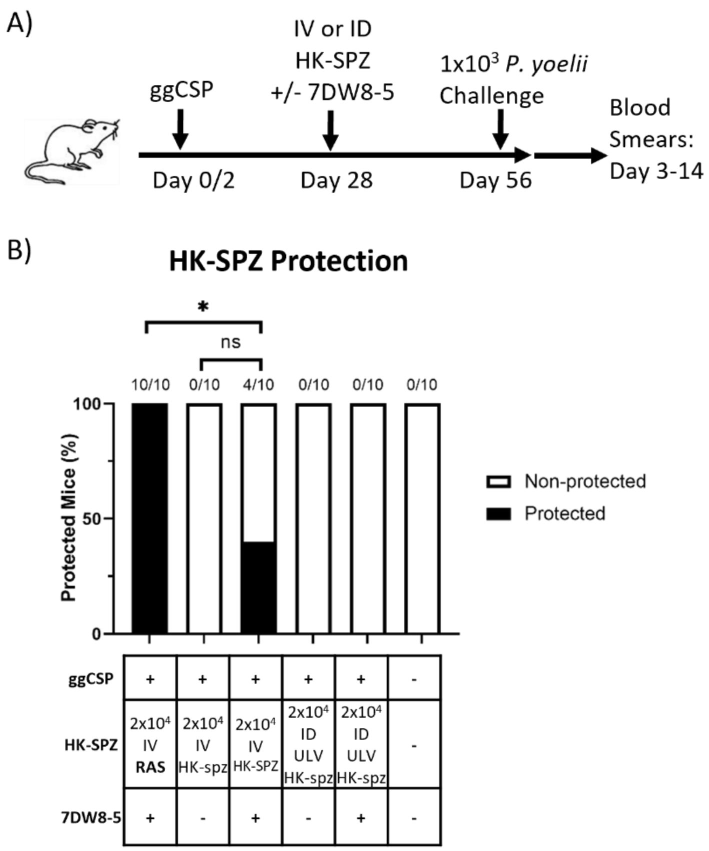

Supplementary Figure 4: HK-spz with or without 7DW8-5 are not protective in prime-and-trap

A) Experimental design of prime-and-trap protection studies.

B) Results of protection studies after challenge with $1x10^3$ WT purified Py spz administered four weeks after trapping with $2x10^4$ HK-spz +/- 7DW8-5 administered IV or ID ULV (2.5 µL, X2 injections). $2x10^4$ IV RAS+7DW8-5 was used as positive control. Protection data from N=10 mice across two independent experiments and was analyzed with Fisher Exact Test, *p<0.05, ns p>0.05.

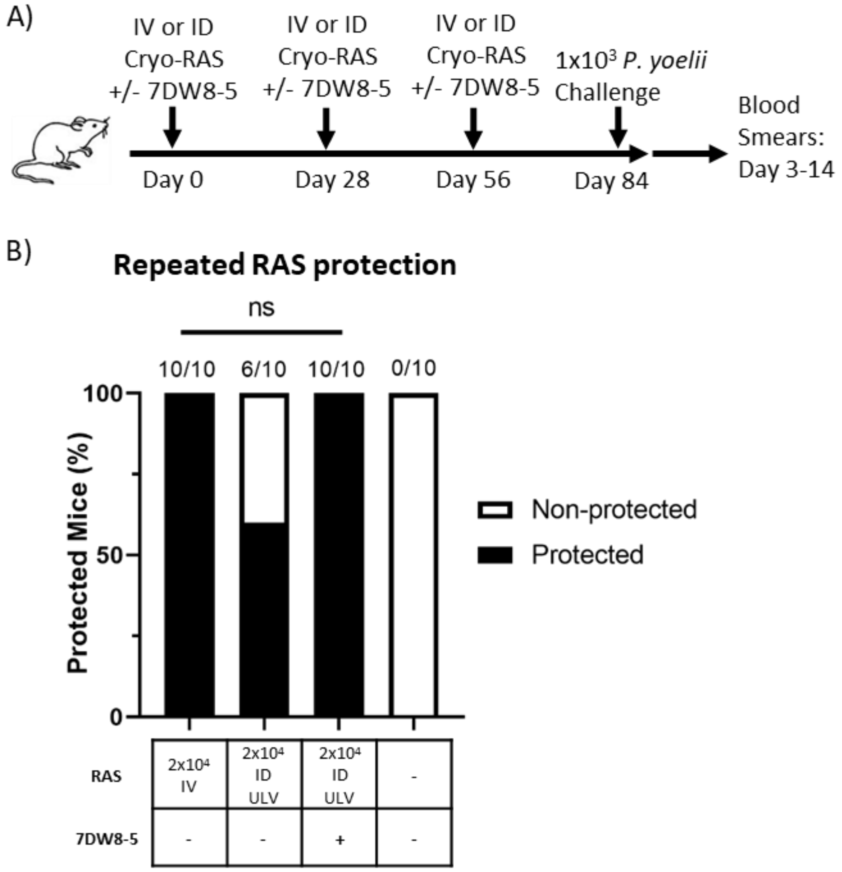

Supplementary Figure 5: 7DW8-5 potentiates ultra-low volume repeated ID-RAS only vaccination

A) Experimental design of repeated RAS protection studies.

B) Results of protection studies after challenge with $1x10^3$ WT purified Py spz administered four weeks after final RAS immunization. RAS was administered IV or ID ULV (2.5 µL, X2 injections) with or without 7DW8-5. Protection data from N=10 mice across two independent experiments and was analyzed with Fisher Exact Test, ns p>0.05 (p-value=0.08).

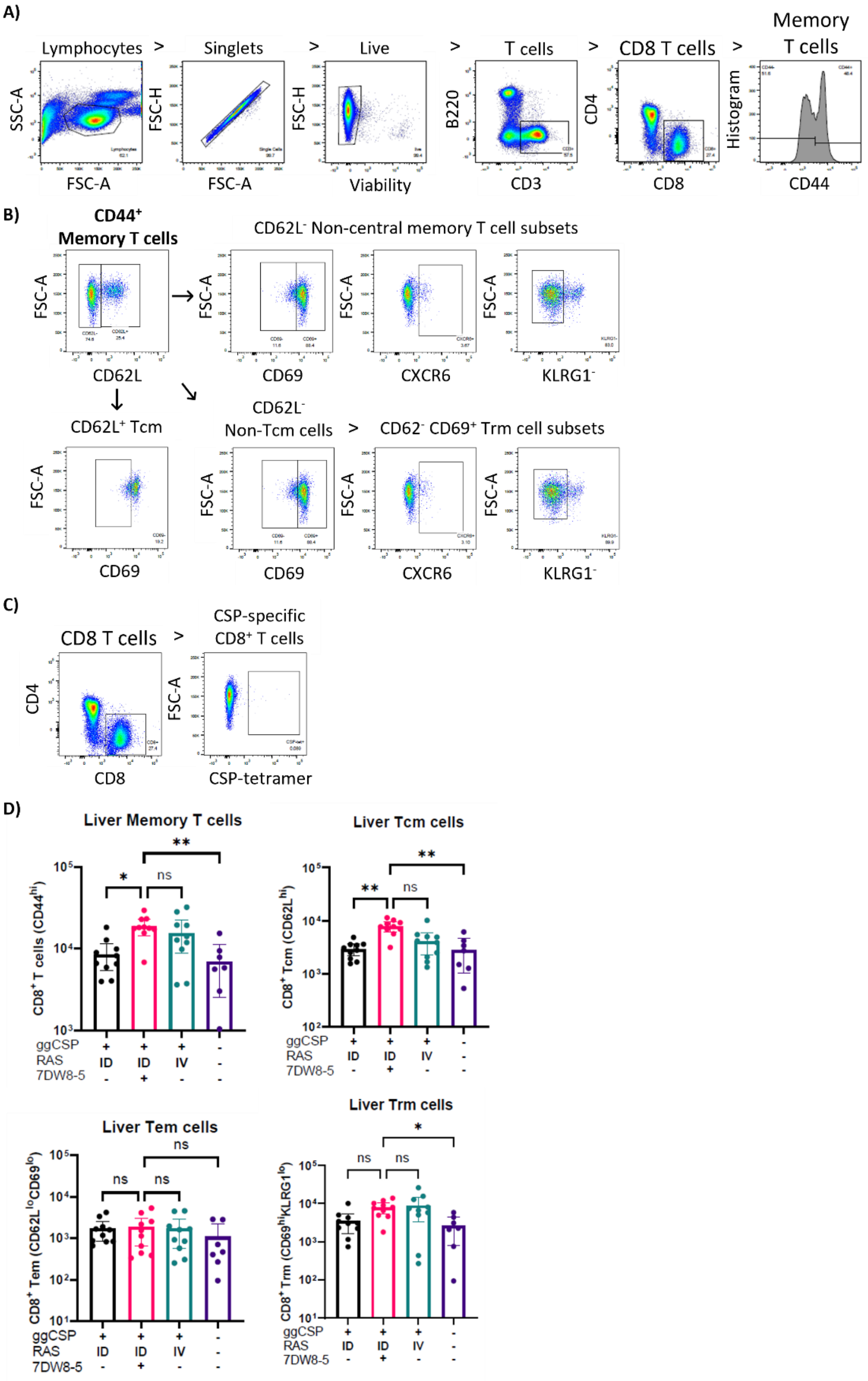

Supplementary Figure 6: Flow cytometry gating strategy for mouse cells

A-B) Flow cytometry liver cell memory T cell gating strategy. One representative animal is shown for all.

C) Flow cytometry liver cell CSP-tetramer gating strategy.

D) Flow cytometry of memory $CD8^+$ T cell subsets from Figure 2 livers. Memory T cell subsets: Overall memory T cells, Tissue central memory (Tcm), Tissue effector memory (Tem), Tissue resident memory (Trm).

Error bars represent SD the mean from N=7-10 mice across two experiments. Data was analyzed with Kruskal-Wallis test with Dunn's multiple comparisons, **p<0.01, *p<0.05, ns p>0.05. All ULV ID-RAS injections were 2.5 µL, X2 injections.

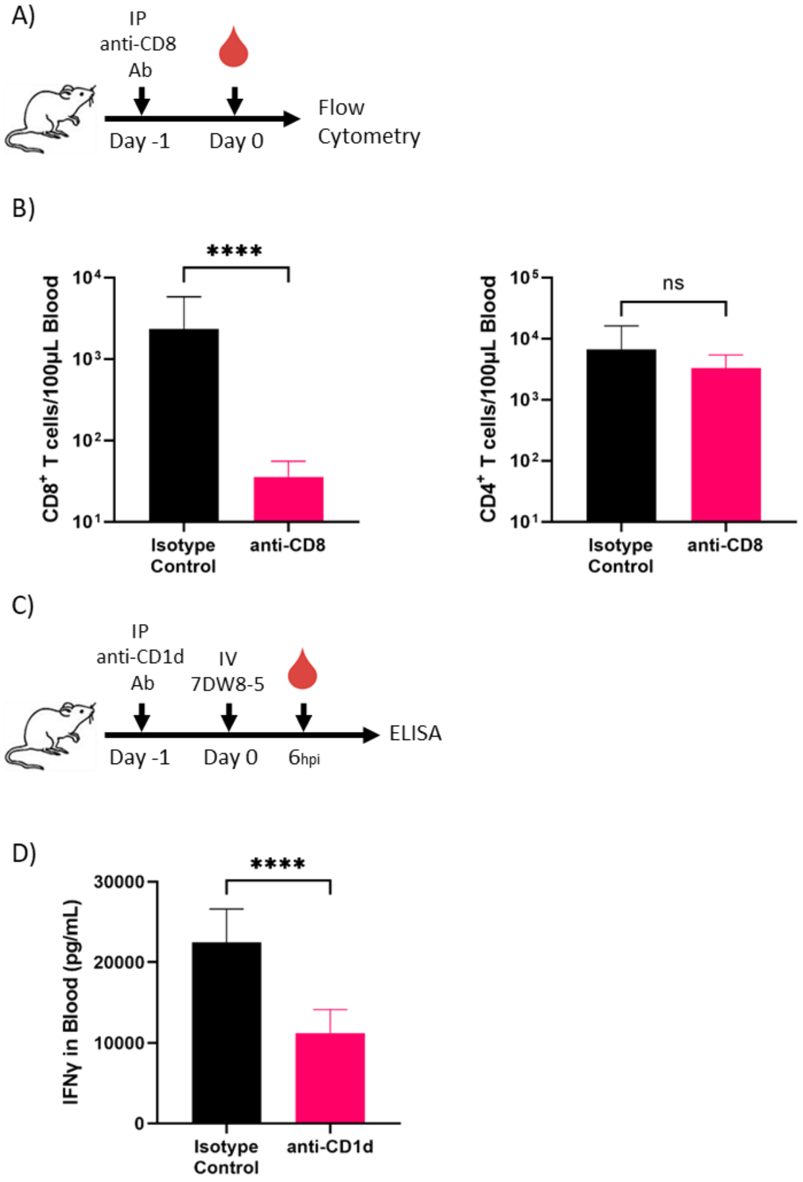

Supplementary Figure 7: Cell depletion/blocking confirmation

A) Experimental design of mouse whole blood leukocyte flow cytometry to confirm CD8 cell depletion at specified schedule and dose.

B) Flow cytometry of leukocytes from (A) mouse blood. Error bars represent SD of N=9 mice from one experiment. Mann–Whitney test, ****p<0.0001, ns p>0.05.

C) Experimental design of mouse blood plasma ELISA studies to confirm CD1d cell blocking at specified schedule and dose.

D) IFN-γ cytokine levels in plasma from (C) mouse blood. Error bars represent SD of N=10 mice from one experiment. Unpaired T test, ****p<0.0001.

A)

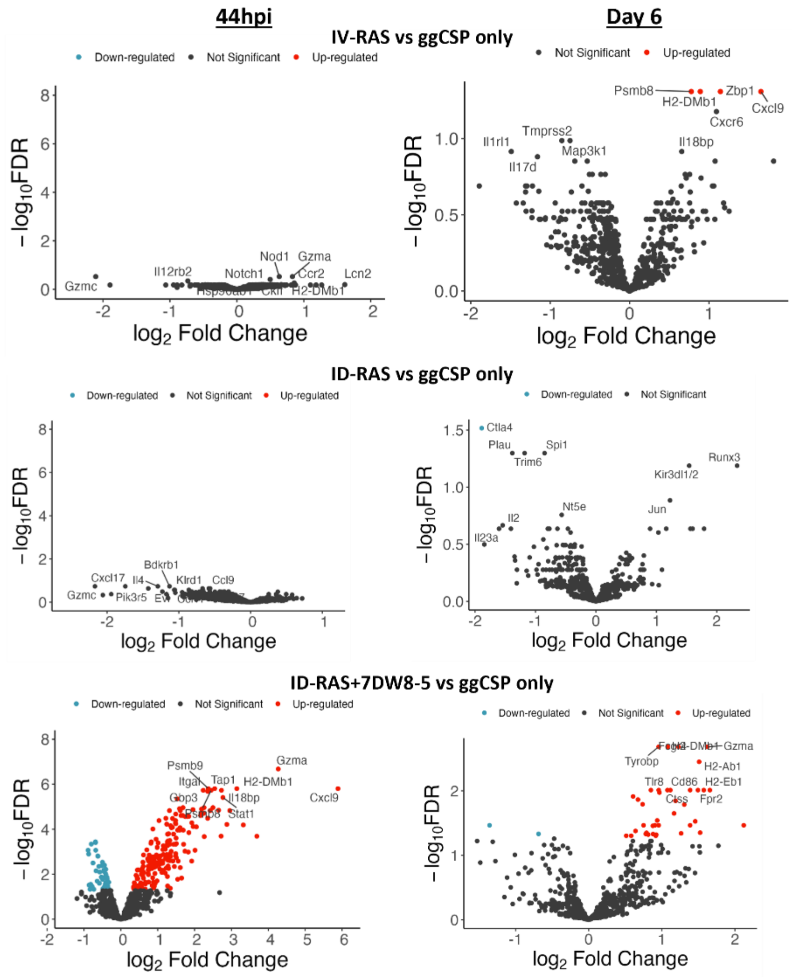

B)

RAS Trap Group (44hpi)	Up	Down	Log_2FC	FDR P-value
IV-RAS vs ID-RAS	17	0	≥0.8	≤0.1
IV-RAS vs ggCSP	0	0	≥0.8	≤0.1
ID-RAS vs ggCSP	0	0	≥0.8	≤0.1
ID-RAS+7DW8-5 vs ggCSP	119	0	≥1	≤0.05
ID-RAS+7DW8-5 vs ID-RAS	154	0	≥1	≤0.05
ID-RAS+7DW8-5 vs IV-RAS	106	0	≥1	≤0.05

RAS Trap Group (Day 6)	Up	Down	Log_2FC	FDR P-value
IV-RAS vs ggCSP	7	4	≥0.8	≤0.1
ID-RAS vs ggCSP	3	4	≥0.8	≤0.1
ID-RAS+7DW8-5 vs ggCSP	22	1	≥1	≤0.05
ID-RAS+7DW8-5 vs ID-RAS	29	1	≥1	≤0.05
ID-RAS+7DW8-5 vs IV-RAS	2	0	≥1	≤0.05

C)

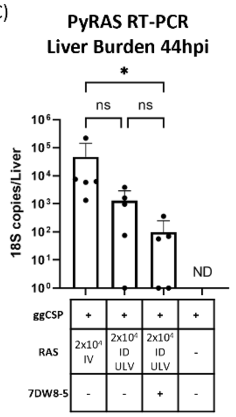

Supplementary Figure 8: Nanostring differentially expressed genes and parasite liver burden

A) Volcano plots of Figure 3F liver transciptomic data. Livers were harvested at 44hpi (left) or Day 6 post injection (right). Only significant genes are highlighted. Significance is defined as FDR Adj. P≤0.05 and log2 fold change of ±1.

B) Differentially expressed genes (DEGs) for indicated groups.

C) Livers from Figure 3F were harvested 44 hours after trapping with cryo-RAS IV (100 µL) or ID ULV (2.5 µL, X2 injections) with or without 7DW8-5 and subjected to Nanostring analysis (Figure 3) or RT-PCR liver burden analysis. 18S pan *Plasmodium* primers were utilized to measure parasite liver burden. RT-PCR data are shown as absolute 18S rRNA copy numbers based on absolute calibrator. Liver sample error bars represent SD of mean of N=5 mice from one experiment. Data was analyzed with Kruskal-Wallis test with Dunn's multiple comparisons, *p<0.05, ns p>0.05.

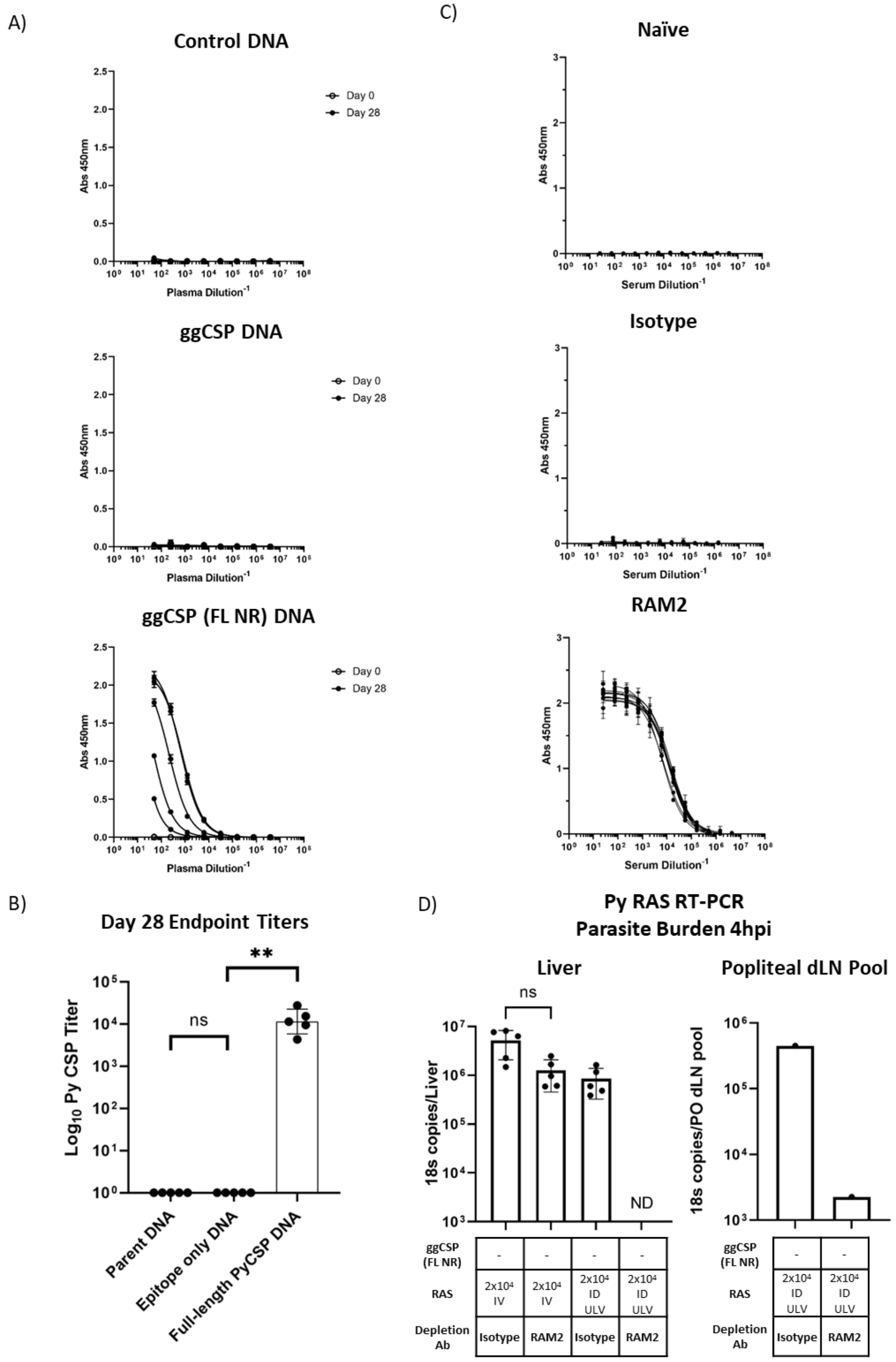

Supplementary Figure 9: PyCSP antibodies impact spz vaccination

A) Mice were primed with ggCSP, ggCSP (FL NR), or control DNA (pUb.3 plasmid backbone without the CSP insert). Serum was isolated from mouse blood on Day 0 and Day 28 for CSP ELISA analysis. Data reported as anti-CSP IgG titers.

B) Day 28 CSP ELISA endpoint titers from (A) mice. Error bars represent SD of mean of N=5 mice from one experiment. ELISA data was analyzed with Kruskal-Wallis test with Dunn's multiple comparisons, **$p<0.01$, ns $p>0.05$.

C) Anti-RAM2 serum direct ELISA dilutions from a subset of isotype, RAM2, or naïve mice from Figure 5 mice.

D) Naïve mice were injected with 150µg RAM2 or isotype control mAb IP. 24 hours later, mice were immunized with cryo-RAS IV (100 µL) or ID ULV (2.5 µL, X2 injections). Four hours post injection livers (left), and popliteal draining lymph nodes (PO dLN) (right) were excised and processed for real-time reverse transcription polymerase chain reaction (RT-PCR) to measure parasite burden with18S pan *Plasmodium* primers. Error bars represent the SD of the mean of N=5 mice from one experiment. PO dLN samples were collected from the injected side of ID-RAS animals and samples were processed in a pool of N=5 mice. Data was analyzed with Kruskal-Wallis test with Dunn's multiple comparisons, ns $p>0.05$. ND=Not Detected.

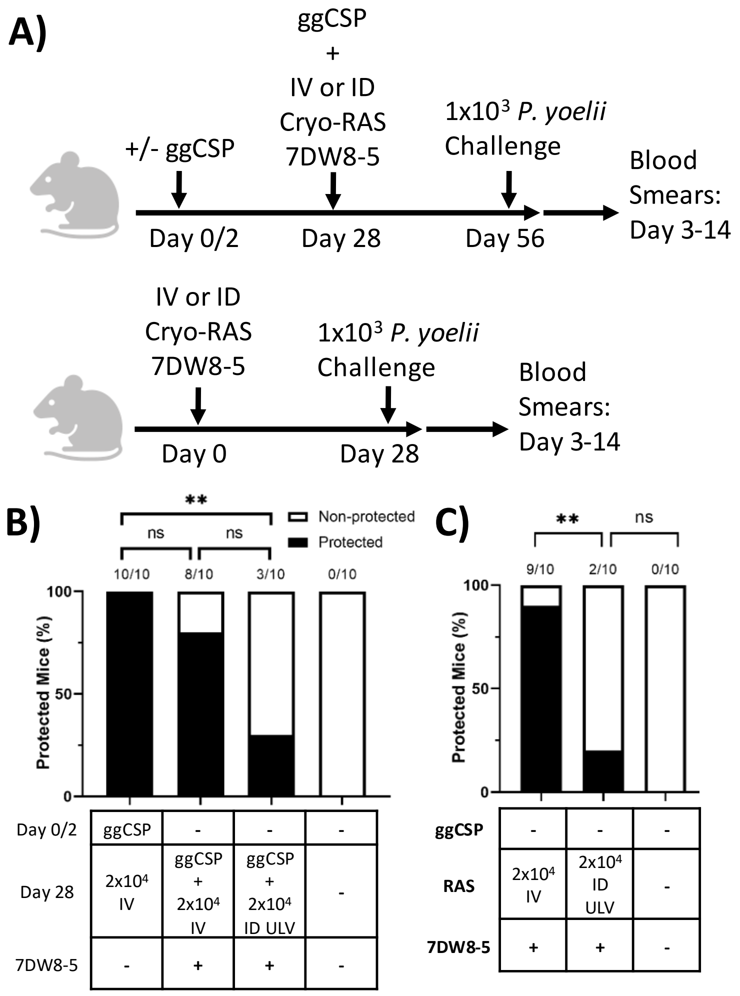

Supplementary Figure 10: Single-day condensed prime-and-trap is protective with IV RAS+7DW8-5

A) Experimental design of standard or single-day condensed prime-and-trap protection studies. For single-day condensed prime-and-trap groups, mice were gene gun primed with two cartridges (0.5 µg DNA per cartridge) immediately followed by RAS+7DW8-5 administration.

B-C) Results of protection studies after challenge with $1x10^3$ WT purified Py spz administered four weeks after RAS trapping. Protection data was analyzed with Fisher Exact test, **p<0.01, ns=p>0.05 from N=10 mice across two independent experiments.
